# Neonatal administration of *Lactiplantibacillus plantarum* ATCC 202195 with or without fructooligosaccharide in Bangladesh: a placebo-controlled randomized trial

**DOI:** 10.1128/msphere.01032-24

**Published:** 2025-02-24

**Authors:** Lisa G. Pell, Huma Qamar, Diego G. Bassani, Cole Heasley, Celine Funk, Chun-Yuan Chen, Jakaria Shawon, Karen M. O'Callaghan, Eleanor Pullenayegum, Davidson H. Hamer, Rashidul Haque, Mamun Kabir, Tahmeed Ahmed, Ciobha O'Kelly, Md Iqbal Hossain, Afreen Z. Khan, Miranda G. Loutet, Mohammad Shahidul Islam, Shaun K. Morris, Prakesh S. Shah, Philip M. Sherman, Shamima Sultana, Abdullah Al Mahmud, Samir K. Saha, Shafiqul A. Sarker, Daniel E. Roth

**Affiliations:** 1 Centre for Global Child Health, Hospital for Sick Children, Toronto, Ontario, Canada; 2 Child Health Evaluative Sciences, Hospital for Sick Children, Toronto, Ontario, Canada; 3 Dalla Lana School of Public Health, University of Toronto 7938 , Toronto, Ontario, Canada; 4 Nutrition Research Division, International Centre for Diarrhoeal Disease Research, Bangladesh, Dhaka, Bangladesh; 5 Department of Nutritional Sciences, King’s College London, London, United Kingdom; 6 Department of Global Health, Boston University School of Public Health and Chobanian & Avedisian School of Medicine, Boston, Massachusetts, USA; 7 Department of Paediatrics, Faculty of Medicine, University of Toronto, Toronto, Ontario, Canada; 8 Child Health Research Foundation 576430 , Dhaka, Bangladesh; 9 Department of Pediatrics, Mt. Sinai Hospital, Toronto, Ontario, Canada; 10 Cell Biology Program, Hospital for Sick Children, Toronto, Ontario, Canada; University of Wyoming College of Agriculture Life Sciences and Natural Resources, Laramie, Wyoming, USA

**Keywords:** probiotic, synbiotic, lactobacilli, neonate, safety, tolerability, colonization, severe infection, developing countries, randomized controlled trial

## Abstract

*Lactiplantibacillus plantarum* ATCC 202195 (LP202195) plus fructooligosaccharide (FOS) for 7 days was previously shown to colonize the infant intestine up to 6 months of age and reduced sepsis rates among young infants in rural India. In a phase 2 randomized controlled trial in Dhaka, Bangladesh (*N* = 519), neonatal administration of LP202195 for 1 or 7 days, with or without FOS, increased LP202195 stool abundance from 14 to 60 days of age, versus placebo. Abundance progressively declined in the post-administration period and did not persist beyond 2 months of age. FOS did not affect LP202195 abundance or its duration of persistence. All regimens were well-tolerated and safe. The absence of LP202195 colonization was inconsistent with results from a prior trial. Additional large-scale trials of LP202195 ± FOS are needed to establish its efficacy in infants who do not become LP202195-colonized.

**IMPORTANCE:**

Among infants born in Dhaka, Bangladesh, a 7-day regimen of *Lactiplantibacillus plantarum* ATCC 202195 (LP202195) plus fructooligosaccharide (FOS) did not colonize the infant gastrointestinal (GI) tract. The absence of colonization is inconsistent with a prior study of the same synbiotic regimen in India, in which LP202195 was shown to persist in the infant GI tract for up to 6 months. Sustained LP202195 colonization was thought to be required for the probiotic to impart its beneficial impact on newborn sepsis. Therefore, additional trials are warranted to confirm the previously observed effects of LP202195 on infant clinical outcomes in the absence of LP202195 colonization. Moreover, since regimens of LP202195 that did not include FOS were indistinguishable from the synbiotic in terms of colonization, safety, and tolerability, future trials should assess the role of FOS for clinical efficacy; removing FOS would reduce costs, an important consideration for scale-up.

**CLINICAL TRIALS:**

This study has been registered at ClinicalTrials.gov as NCT05180201.

## INTRODUCTION

Probiotics have been found in numerous studies to reduce the risk of morbidities in preterm infants, including necrotizing enterocolitis (1, 2) and late-onset sepsis (3), as well as decrease mortality (2). While most probiotic trial data have been generated in preterm very low birthweight populations (1–3), and relatively few probiotic trials have been conducted in low- and middle-income countries (LMICs) (4), a prominent exception was the 2017 publication of findings of a placebo-controlled randomized trial in rural India in which 4,556 near- and full-term newborns received either a 7-day oral regimen of *Lactiplantibacillus plantarum* ATCC 202195 (LP202195) plus fructooligosaccharide (FOS) or placebo (maltodextrin) beginning at days 2 to 4 after birth (5). In the LP202195 + FOS group, there was a 42% reduction in the incidence of sepsis within the first 60 days after birth, compared to placebo, as well as reduced rates of culture-positive and culture-negative sepsis, and lower respiratory tract infections, compared to placebo (5). *L. plantarum* was not isolated from any blood cultures, and very few gastrointestinal (GI)-related adverse events were documented (5). However, additional evidence regarding the use of LP202195 in other LMIC settings is required before implementing and scaling-up a 7-day regimen of LP202195 + FOS to reduce newborn sepsis in LMICs.

The effect of LP202195 + FOS on colonization of the infant GI tract was not reported in the above-mentioned community-based trial in India (5). However, a prior smaller trial (*n* = 31) led by the same principal investigator in a hospital setting in India showed that among infants administered the same 7-day regimen of LP202195 + FOS, *L. plantarum* was detected starting 3 days after administration of the first synbiotic dose, and remained present in all stool samples at 2 months of age, persisting for at least 6 months after the administration of the last dose in samples collected from one-third of the infants (6). Conclusions regarding probiotic colonization may not be generalizable because colonization is influenced by gut microbiota composition (7), which varies by geographic setting (8), mode of delivery (9, 10), and other maternal and newborn characteristics (9, 11). Since all infants in the hospital-based trial of LP202195 (6) were delivered via C-section, whereas less than 1% of the babies enrolled in the community-based trial of LP202195 were delivered via C-section, clinical effects, as observed in the community-based trial of LP202195, may differ in settings with higher rates of C-section (5).

As neither of the two previously conducted trials of LP202195 included a probiotic-only intervention group or groups with different synbiotic administration schedules, it was unknown whether a 7-day course of LP202195 alone, without the addition of FOS, or shorter regimens of LP202195, with or without FOS, would have resulted in similar outcomes. A 7-day regimen without FOS and/or a shorter regimen, if found comparable to the 7-day probiotic plus FOS regimen, would substantially lower the cost per recipient and may increase the feasibility of implementation in LMICs, particularly if a shorter regimen is used.

Here, we report the results of a phase 2 randomized, placebo-controlled, double-blinded, five-group trial of neonatal oral administration of LP202195 (10^9^ colony forming units [CFU]/day). We estimated the effect of administering LP202195, with or without FOS, beginning on days 0 to 4 after birth, for 1 or 7 days, versus placebo (maltodextrin), on the absolute stool abundance of LP202195, using strain-specific qPCR (12), and on several safety and tolerability endpoints among generally healthy infants in Dhaka, Bangladesh.

## RESULTS

### Description of trial participants and adherence to the investigational products

**Fig 1:**
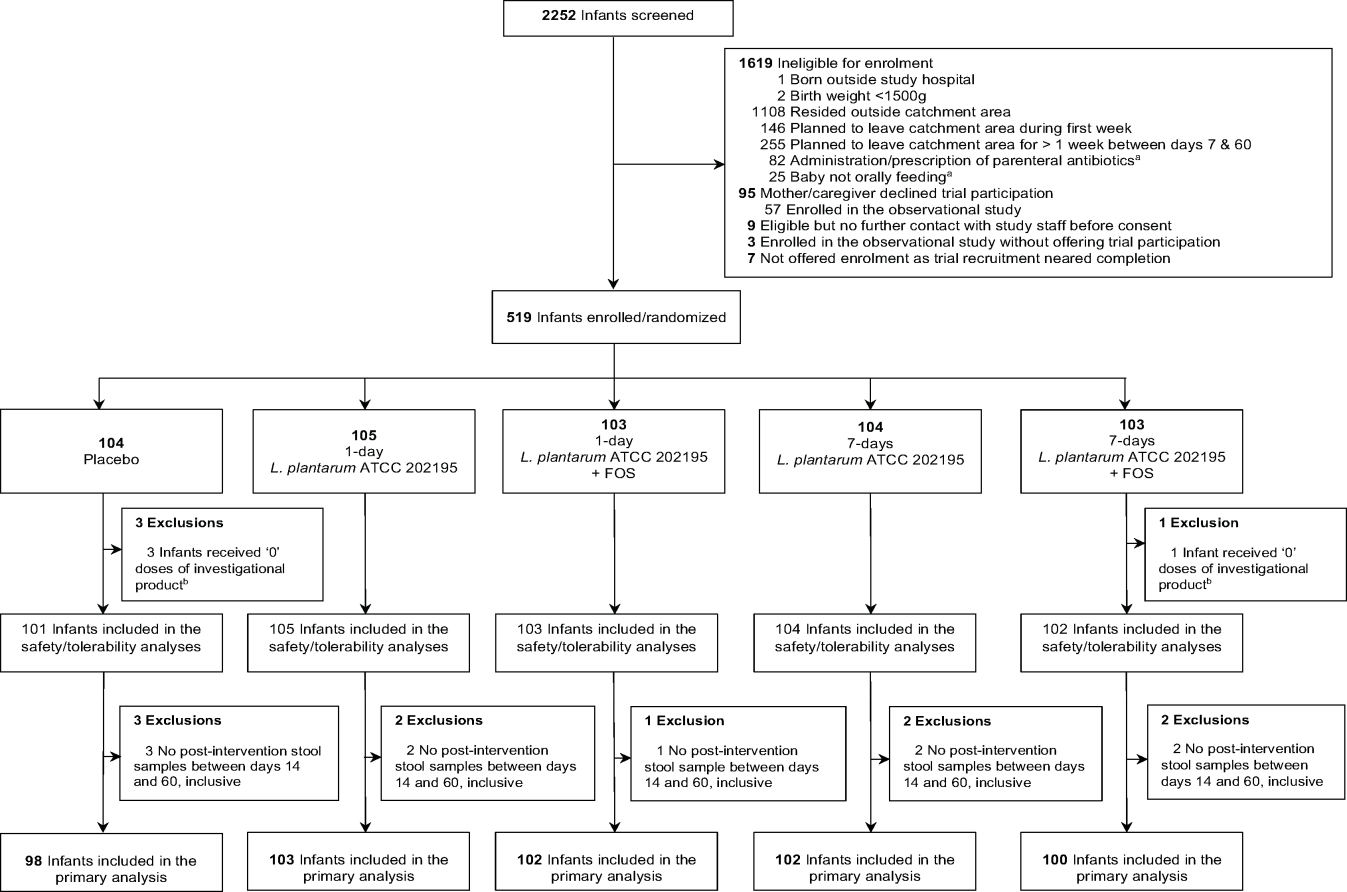
Trial CONSORT flow diagram. ^a^Exclusions due to the administration or prescription of parenteral antibiotics or due to neonates not orally feeding were based on criteria documented at the last available screening visit, irrespective of the infant’s age when they were last screened. ^b^All infants who received zero doses of investigational product also had zero post-intervention stool samples collected between days 14 and 60, inclusive, and thus were not included in the safety and tolerability analyses or in the primary outcome analysis.

Between 7 January 2022 and 24 April 2022, a total of 519 young infants between 0 and 4 days of age were enrolled and randomized into one of the five groups: placebo; a 1-day regimen of LP202195 with or without FOS (LP1 + FOS or LP1, respectively); or a 7-day regimen of LP202195 with or without FOS (LP7 + FOS or LP7, respectively) (Fig. 1). Eligible infants were delivered at one of two study-associated public hospitals in Dhaka City (Maternal and Child Health Training Institute [MCHTI] or Mohammadpur Fertility Services and Training Centre [MFSTC]), weighed at least 1,500 g at birth, and were orally feeding at the time of eligibility assessment, and with the caregiver intending to maintain residence within the study catchment area until the infant was 60 days of age. Infants who were severely ill or receiving parenteral antibiotics at the time of screening, among other exclusion criteria, were not eligible for participation (details in Materials and Methods). Most participants (~75% in each group) were enrolled at the MFSTC site. Baseline characteristics were similar across intervention groups (Table 1) but differed between enrollment hospitals (Table S1). Intervention groups had similar proportions of routine baseline visits at which a clinical sign was observed by a medical officer (Table S2). Adherence to the investigational product (IP) was similar across intervention groups. Most infants (≥93% in each intervention group) received all seven IP doses by 10 days of postnatal age. The median age at first and last IP dose was 2 and 8 days, respectively, across all trial arms (Table S3). The majority of successfully administered IP doses (76%–80% in each group) were dissolved in human milk (Table S4). Given the observed differences in baseline characteristics between enrollment hospitals, unless specified otherwise, all further analyses were adjusted for enrollment hospital.

**TABLE 1:** Maternal, household, delivery, and infant characteristics by intervention group

Parameter	Intervention group					
	Placebo	LP1	LP1 + FOS	LP7	LP7 + FOS	
Participants, *N*	104	105	103	104	103	
Maternal age (years), median (25th, 75th)	23 (20, 26.5)	23 (20, 26)	25 (20, 27)	22 (20, 27)	23 (20, 26)	
Maternal education, *n* (%)						
Little to no schooling^*a*^	22 (21)	35 (33)	37 (36)	32 (31)	30 (29)	
Secondary school incomplete	37 (36)	29 (28)	27 (26)	32 (31)	30 (29)	
Secondary school complete or higher	45 (43)	41 (39)	39 (38)	40 (38)	43 (42)	
Gravidity, median (25th, 75th)	2 (1, 3)	2 (1, 3)	2 (1, 3)	2 (1, 3)	2 (1, 2)	
First pregnancy, *n* (%)	41 (39)	38 (36)	37 (36)	35 (34)	45 (44)	
First live birth, *n* (%)	49 (47)	48 (46)	42 (41)	47 (45)	51 (50)	
Gestational age at delivery (weeks),^*b*^ median (25th, 75th)	39.1 (38.1, 40.3)	39.0 (38.0, 40.1)	38.9 (37.9, 39.9)	39.0 (38.3, 39.9)	39.1 (38.3, 39.9)	
Term (≥37 weeks), *n* (%)	96 (95)	93 (90)	94 (92)	96 (93)	91 (91)	
Preterm (<37 weeks), *n* (%)	5 (5.0)	10 (9.7)	8 (7.8)	7 (6.8)	9 (9.0)	
Hospital enrollment site, *n* (%)						
MFSTC	78 (75)	78 (74)	76 (74)	78 (75)	77 (75)	
MCHTI	26 (25)	27 (26)	27 (26)	26 (25)	26 (25)	
Mode of delivery, *n* (%)						
Vaginal	58 (56)	54 (51)	57 (55)	52 (50)	56 (54)	
C-section	46 (44)	51 (49)	46 (45)	52 (50)	47 (46)	
Maternal peripartum antibiotics administered,^*c*^ *n* (%)	102 (98)	103 (98)	102 (99)	101 (97)	97 (94)	
Asset index quintile,^*d*^ *n* (%)						
1 (lowest)	24 (23)	23 (22)	18 (17)	19 (18)	25 (24)	
2	22 (21)	23 (22)	21 (20)	20 (19)	17 (17)	
3	20 (19)	28 (27)	19 (18)	27 (26)	23 (22)	
4	14 (13)	16 (15)	28 (27)	16 (15)	23 (22)	
5 (highest)	24 (23)	15 (14)	17 (17)	22 (21)	15 (15)	
Infant age at enrollment (days), median (min, max)	1 (0, 4)	1 (0, 4)	1 (0, 4)	1 (0, 4)	1 (0, 4)	
Sex, *n* (%)						
Male	49 (47)	67 (64)	60 (58)	52 (50)	52 (50)	
Female	55 (53)	38 (36)	43 (42)	52 (50)	51 (50)	
Birth weight (g),^*e*^ mean (SD)	2,893 (366)	2,898 (355)	2,905 (387)	2,894 (356)	2,853 (359)	
Feeding pattern at or near enrollment,^*f*^ *n* (%)						
Exclusively breastfed	84 (82)	92 (88)	87 (84)	88 (85)	94 (91)	
Not exclusively breastfed or not breastfed	19 (18)	13 (12)	16 (16)	16 (15)	9 (8.7)	

^*a*^ Includes women with no formal education, and incomplete and completed primary school.

^*b*^ Gestational age data missing for 10 infants: 3 in placebo, 2 in LP1, 1 in LP1 + FOS, 1 in LP7, 3 in LP7 + FOS.

^*c*^ Peripartum period refers to antibiotics that were administered in the hospital during labor and/or in the operating theater and/or after delivery, up to and including 4 days postpartum.

^*d*^ Asset index scores and quintiles were generated using principal components analysis for all participants enrolled in the trial (*n* = 519) and in a concurrent observational study (*n* = 1886) at the same study sites with the same eligibility criteria. Scores represent a summary measure of household wealth based on ownership of the following items: electricity, fan, mobile, almirah, fridge, television, chair, table, watch, bicycle, computer, freezer, pump, vehicle, rickshaw, phone, radio, autobike, cats, birds, poultry, dogs, goats, cows, and other animals. Lower scores reflect ownership of fewer items (i.e., lower wealth) and higher scores reflect the ownership of more items (i.e., higher wealth).

^*e*^ Weight measured by study personnel was used as a birthweight proxy for four infants where there was >15% difference between birth weight and weight measured within 1 to 4 days of birth; for three infants, weight was measured within 1 day of birth and for one infant, weight measured within 4 days of birth was used.

^*f*^ Feeding pattern was ascertained using data collected at the first routine clinical visit after the first IP dose, or at the earliest available routine clinical visit if the infant did not receive IP. Otherwise, feeding data were derived during data collection at the baseline visit. Prelacteal feeds were not considered when ascertaining feeding patterns. Feeding data were missing for one infant in the placebo group.

### LP202195 with or without FOS, for 1 or 7 days, increased the average absolute abundance of LP202195 in post-intervention stool samples between 14 and 60 days of age

Stool samples from 505 infants were collected in the post-intervention period between days 14 and 60 (post_14-60_) (Fig. 1; Table S5). Most infants provided two (39%) or three (35%) stool samples during this period, which were analyzed by qPCR, and the number of samples analyzed per infant was evenly distributed across intervention groups (Table S5). LP202195 was detected via qPCR in 4% of the post_14-60_ stool samples among infants receiving placebo, in 31% of those receiving LP1, in 38% of those receiving LP1 + FOS, in 55% of those receiving LP7, and 62% of those receiving LP7 + FOS (Fig. S1).

The mean absolute abundance (AA) of LP202195 (cells/µg DNA) was higher in all intervention groups during the post_14-60_ period compared to placebo (Table 2). These findings were robust to alternative derivations of AA (e.g., log_10_ cells/gram of stool, log_10_ cells, etc.), alternate time periods of stool sample collection, and in per-protocol (Table S6) and sub-group analyses (Table S7). The mean differences in AA of LP202195 between each intervention group versus placebo also did not significantly differ between modes of delivery (Table S7). During the post_14-60_ period, there was an increased relative probability of samples having a high AA of LP202195, defined as having 3 or more log_10_ cells/µg DNA of LP202195, compared to samples with a low AA of LP202195 (less than 3 log_10_ cells/µg DNA of LP202195), among infants receiving the IP containing LP202195, compared to placebo (Table 2).

**TABLE 2:** Effect of administration of LP202195 with or without FOS on the absolute abundance of LP202195 in stool samples collected post-intervention (days 14 to 60), relative to placebo or LP7 + FOS, and relative risk of high abundance^*a*^

Parameter	Intervention group				
	Placebo	LP1	LP1 + FOS	LP7	LP7 + FOS
Number of samples, *N*	278	267	280	296	272
AA_Post(14-60)_ of LP202195 (log_10_ cells/µg DNA), mean ± SE	1.86 ± 0.03	2.38 ± 0.05	2.58 ± 0.07	3.09 ± 0.07	3.15 ± 0.06
Mean difference, relative to placebo, in AA of LP202195 (log_10_ cells/µg DNA), (95% CI)	Ref	0.53 (0.41, 0.65)	0.73 (0.59, 0.88)	1.24 (1.09, 1.38)	1.30 (1.16, 1.43)
Mean difference, relative to LP7 + FOS, in AA of LP202195 (log_10_ cells/µg DNA), (95% CI)	NA	−0.77 (−0.93, –0.60)	−0.56 (−0.74, –0.38)	−0.06 (−0.24, 0.12)	Ref
Samples in low abundance group (i.e., AA < 3 log_10_ cells/µg DNA), *n* (%)	269 (97)	204 (76)	203 (72)	167 (56)	139 (51)
Samples in high abundance group (i.e., AA ≥ 3 log_10_ cells/ µg DNA), *n* (%)	9 (3.2)	63 (24)	77 (28)	129 (44)	133 (49)
Relative risk (RR) of high (vs low) abundance, relative to placebo (95% CI)	Ref	7.1 (4.3, 11.9)	8.3 (5.0, 13.7)	13.4 (8.2, 22.0)	14.2 (8.7, 23.3)

^*a*^ AA, absolute abundance; NA, not applicable.

### FOS did not affect the absolute stool abundance of LP202195, and the abundance of LP202195 was lower in both 1-day regimen groups compared to the 7-day LP202195 + FOS group

Based on a pre-specified non-inferiority margin of −1 log_10_ cells/µg DNA, all active IP-containing groups were non-inferior to the LP7 + FOS group (Table 2), and these findings were unchanged in pre-specified sensitivity and supplementary analyses where we modified the derivation of AA, the time window of stool sample collection, and the population being analyzed based on adherence to the IP, and various sub-groups (Tables S6 and S8). The mean AA of LP202195 (cells/µg DNA) in post_14-60_ samples was significantly lower in the LP1 + FOS and LP1 groups compared to the LP7 + FOS group, but there was no statistically significant difference in AA between samples from infants who received the IP without FOS versus with FOS for 7 days (Table 2).

### Stool abundance of LP202195 progressively declined beyond the period of administration of LP202195, with or without FOS, suggesting that LP202195 did not colonize the infant GI tract

**Fig 2:**
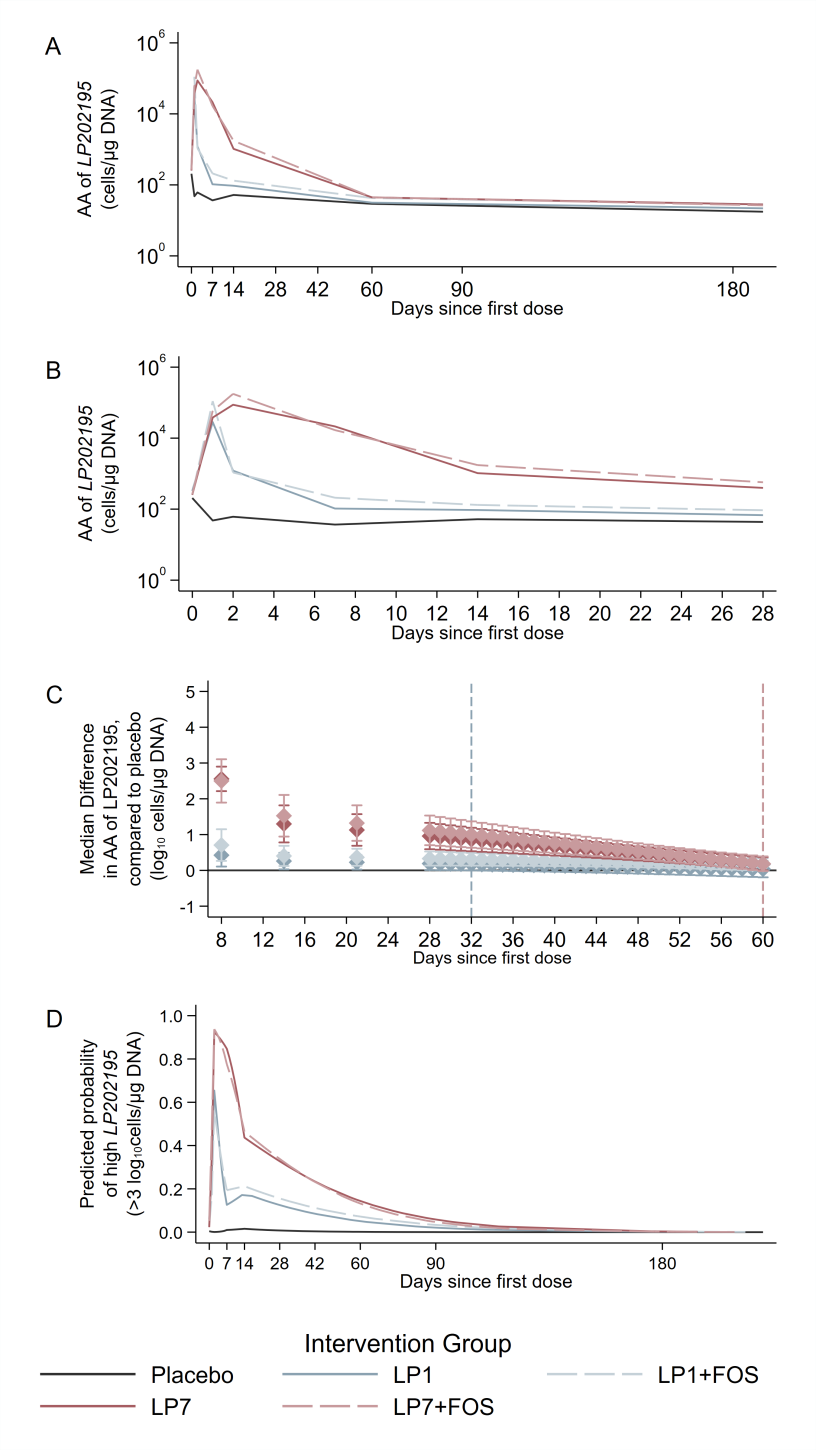
*L. plantarum* ATCC 202195 (LP202195), when administered to neonates in Dhaka, Bangladesh, for 1 or 7 days, with or without FOS, does not result in LP202195 colonization. (**A**) Longitudinal trajectories of LP202195 AA (cells/µg DNA) modeled as a function of days since first IP dose, up to 180 days since first dose, and (B) zooming in on the trajectories within the first 28 days since first IP dose. (**C**) Differences in AA of LP202195 (log_10_ cells/µg DNA) across intervention groups, relative to the placebo group, are shown at discrete time points between 8 and 60 days since first IP dose. The vertical dashed blue line at 32 days since first dose indicates the time point at which the AA in the LP1 group was no longer significantly different from the AA in the placebo group. The vertical dashed red line at 60 days since first dose indicates the time point at which the AAs LP1 + FOS, LP7, and LP7 + FOS groups were no longer significantly different from the AA in the placebo group. (**D**) Predicted probabilities of high versus low AA of LP202195, by days since first dose. In each panel, the placebo group is shown with a black solid line, the LP1 group with the light blue solid line, the LP1 + FOS group with a dashed light blue line, the LP7 group with a solid red line, and the LP7 + FOS group with a dashed red line.

The trajectories of the median AA of LP202195 in the two LP7 groups (with or without FOS) visually differed from the trajectories of the median AA of LP202195 in the two LP1 groups (with or without FOS) (Fig. 2A and B; Fig. S2A and B). In all groups receiving the active IP, average AA of LP202195 peaked between 1 and 2 days after administration of the first dose and declined in the following days regardless of the duration of IP administration. The peak median AA was 10^5.25^ cells/µg DNA and was reached in the LP7 + FOS group 2 days after the first dose, closely followed by the LP1 + FOS group (10^5.04^) 1 day after administration (Fig. 2B; Fig. S2B). The decay of AA began 1 day after the first dose among infants receiving the active IP for 1 day and following the 2nd day of IP administration among those receiving active IP for 7 days. Among samples collected from infants in the two 7-day groups, the decline in AA was observed during the IP administration period (Fig. 2A and B; Fig. S2A and B). The median AA in all active IP-containing groups was indistinguishable from that observed among samples collected from infants receiving placebo by about 60 days since first dose (Fig. 2A and C; Fig. S2A and C; Table S9). Predicted probabilities of high AA were <0.5 by 22 and 5 days since first dose in the two LP7 groups and two LP1 groups, respectively, and ≤0.3 and ≤0.2 in the two LP7 and two LP1 groups, respectively, at any time point between 60 and 180 days after the first IP dose (Fig. 2D; Fig. S2D). Among all samples used in the longitudinal mixed-effects model with child-specific random intercepts, after adjusting for days since first dose, intervention group, and enrollment hospital, only 20% (95% CI: 17%, 24%) of the total variance in AA was attributable to between-infant variation. While there is no standard definition of probiotic colonization, we considered that colonization of the GI tract would have instead resulted in a persistently elevated steady-state AA of LP202195 in stool samples that were collected beyond the probiotic administration period.

### LP202195, when administered to neonates for 1 or 7 days, with or without FOS, was safe and well-tolerated

There were no effects of the intervention on the total number of hospitalization events, deaths, or on the number of hospitalization events associated with GI-related signs, symptoms, or illnesses during the first 180 days after birth. Three infant deaths occurred: two in the LP1 group, and one in the LP7 group. Lactobacilli were not isolated from any blood (*n* = 21) or urine (*n* = 10) samples that were collected in the evaluation of illness episodes. A total of 27 severe infection (SI) events occurred up to 60 days, 9 of which occurred during the intervention period, and there was no effect of the intervention on SI events or on a composite outcome of SIs plus deaths (Table 3). There were no differences in average biochemical analyte concentrations or hematological parameters, or in the number and proportion of samples for which these measurements were outside the reference range across intervention groups among routinely collected blood samples (Tables S10 and S11).

**TABLE 3:** Hospitalizations, deaths, and severe infections by intervention group^*i*^

Parameter	Intervention group				
	Placebo	LP1	LP1 + FOS	LP7	LP7 + FOS
Participants, *N*	104	105	103	104	103
Total hospitalizations during IP period,^*a*^^,*b*^ *n*	7	5	7	8	6
Total hospitalizations up to 60 days,^*c*^ inclusive, *n*	10	9	12	13	10
Total hospitalizations between 61 days and 180 days,^*c*^ inclusive, *n*	1	8	7	3	3
Hospitalizations^*a*^ up to 180 days^*c*^					
Infants with at least one event, *n* (%)	11 (11)	13 (12)	16 (16)	15 (14)	12 (12)
Number of events, *n*	11	17	19	16	13
Person-time at risk (infant-days)	17,824	18,238	18,272	18,082	18,250
Incidence rate (95% CI), events per 1,000 infant-days	0.62 (0.32, 1.1)	0.93 (0.56, 1.4)	1.0 (0.64, 1.6)	0.88 (0.52, 1.4)	0.71 (0.39, 1.2)
Hazard ratio (95% CI)	Ref	1.5 (0.66, 3.3)	1.7 (0.79, 3.5)	1.4 (0.68, 3.0)	1.1 (0.52, 2.5)
Duration of hospitalization (days),^*d*^ median (25th, 75th)	3.9 (3.1, 4.8)	5.9 (2.9, 6.9)	4.6 (2.1, 5.9)	5.1 (3.9, 6.9)	3.8 (3.0, 7.0)
Hospitalizations associated with gastrointestinal (GI) symptoms/signs up to 180 days^*e*^					
Infants with at least one event, *n* (%)	2 (1.9)	5 (4.8)	5 (4.9)	2 (1.9)	2 (1.9)
Number of events, *n*	2	5	5	2	2
Deaths,^*f*^ *n* (%)	0 (0)	2 (1.9)	0 (0)	1 (0.96)	0 (0)
Deaths up to 60 days, *n* (%)	0 (0)	1 (0.95)	0 (0)	1 (0.96)	0 (0)
SIs^*g*^ up to 60 days					
Total up to 60 days,^*c*^ inclusive					
Infants with at least one event, *n* (%)	6 (5.8)	5 (4.8)	5 (4.9)	5 (4.8)	4 (3.9)
Number of events, *n*	6	6	6	5	4
Person-time at risk (infant-days)	5,870	6,043	6,018	5,997	6,035
Incidence rate (95% CI), events per 1,000 infant-days	1.0 (0.41, 2.1)	0.99 (0.39, 2.0)	1.0 (0.40, 2.0)	0.83 (0.30, 1.8)	0.66 (0.21, 1.5)
Hazard ratio (95% CI)^*h*^	Ref	0.97 (0.29, 3.2)	0.96 (0.29, 3.2)	0.82 (0.26, 2.6)	0.64 (0.19, 2.2)
After first IP dose and up to 60 days,^*b*^ inclusive					
Infants with at least one event, *n* (%)	6 (5.9)	5 (4.8)	5 (4.9)	4 (3.8)	4 (3.9)
Number of events, *n*	6	6	6	4	4
During IP period^*a*^^,*b*^					
Infants with at least one event, *n* (%)	3 (3.0)	2 (1.9)	1 (1.0)	3 (2.9)	0 (0)
Number of events, *n*	3	2	1	3	0
# SI events associated with *Lactobacillus spp*.,^*c*^ *n* (%)	0 (0)	0 (0)	0 (0)	0 (0)	0 (0)
Non-injury deaths^*f*^ or SIs					
Total up to 60 days^*c*^					
Infants who died or had at least one SI, *n* (%)	6 (5.8)	5 (4.8)	5 (4.9)	5 (4.8)	4 (3.9)
Number of events, *n*	6	7	6	5	4
Person-time at risk (infant-days)	5,870	6,043	6,018	5,997	6,035
Incidence rate (95% CI), events per 1,000 infant-days	1.0 (0.41, 2.1)	1.2 (0.50, 2.2)	1.0 (0.40, 2.0)	0.83 (0.30, 1.8)	0.66 (0.21, 1.5)
Hazard ratio (95% CI)^*g*^	Ref	1.1 (0.31, 4.1)	0.97 (0.29, 3.2)	0.82 (0.26, 2.6)	0.64 (0.19, 2.2)
After first IP dose and up to 60 days^*b*^					
Infants who died or had at least one SI, *n* (%)	6 (5.9)	5 (4.8)	5 (4.9)	4 (3.8)	4 (3.9)
Number of events, *n*	6	7	6	4	4
During IP period^*a*^^,*b*^					
Infants who died or had at least one SI, *n* (%)	3 (3.0)	2 (1.9)	1 (1.0)	3 (2.9)	0 (0)

^*a*^ From day of the first IP dose up to 3 days after the last dose.

^*b*^ Among infants who received at least one dose of the IP. *N*_Overall_ = 515; *N*_Placebo_ = 101; *N*_LP1_ = 105; *N*_LP1+FOS_ = 103; *N*_LP7_ = 104; and *N*_LP7+FOS_ = 102.

^*c*^ Among all infants, irrespective of number of doses of IP received.

^*d*^ Includes all hospitalizations, including multiple separate hospitalizations of the same infant.

^*e*^ Among infants with at least one event. For infants with more than one event, only the first event was included in this analysis.

^*f*^ All deaths were due to non-injury-related causes.

^*g*^ Severe infection (SI) events up to 60 days included all six cases of hospitalized lower respiratory tract infection (LRTI) events identified in the trial: two in placebo group; one in LP1 group; two in LP1 + FOS group; 0 in LP7 group; and one in LP7 + FOS group.

^*h*^ Cox proportional hazard models adjusting for hospital enrollment site (see text for details).

^*i*^ IP, investigational product; CI, confidence intervals.

There were no effects of the intervention on the proportions of infants ever having had caregiver-reported clinical symptoms, including GI symptoms, during the IP administration period, or the proportion of visits at which clinical signs were observed by study personnel during (Table 4) or after the IP administration period (Table S12). Likewise, the intervention had no effect on the cumulative incidence of caregiver-reported clinical symptoms in the post-IP administration period (Table S12). There were no between-group differences in the number of *ad hoc* medical assessments of non-hospitalized infants, the proportion of infants with at least one *ad hoc* medical assessment, or the cumulative incidence of individual clinical symptoms or signs that were documented at *ad hoc* medical assessments (Table S13).

**TABLE 4:** Adverse events during the investigational product administration period^*a*^ by intervention group

Parameter	Intervention group					*P^b^*
	Placebo	LP1	LP1 + FOS	LP7	LP7 + FOS	
Caregiver-reported symptoms, *n* (%)^*c*^						
Number of participants, *N*	101	105	103	104	102	
Abdominal distension	0 (0)	0 (0)	1 (1.0)	1 (1.0)	1 (1.0)	0.8
Acute diarrhea	1 (1.0)	1 (1.0)	1 (1.0)	0 (0)	0 (0)	0.7
Persistent vomiting (≥3 times in 24 hours)	1 (1.0)	1 (1.0)	2 (1.9)	0 (0)	0 (0)	0.5
Vomiting	2 (2.0)	1 (1.0)	3 (2.9)	1 (1.0)	1 (1.0)	0.8
≥6 hours since last passed urine	0 (0)	2 (1.9)	1 (1.0)	0 (0)	0 (0)	0.5
Red or discharging umbilicus	2 (2.0)	5 (4.8)	1 (1.0)	4 (3.8)	2 (2.0)	0.5
Skin pustules or boil	1 (1.0)	0 (0)	1 (1.0)	1 (1.0)	2 (2.0)	0.7
Unusual skin rash or anything abnormal on skin	0 (0)	1 (1.0)	2 (1.9)	1 (1.0)	1 (1.0)	0.8
Yellowing of skin or eyes	5 (5.0)	7 (6.7)	11 (11)	8 (7.7)	4 (3.9)	0.3
Red/oozing/swollen eyes	3 (3.0)	4 (3.8)	6 (5.8)	1 (1.0)	5 (4.9)	0.4
Sores inside mouth	0 (0)	0 (0)	1 (1.0)	2 (1.9)	0 (0)	0.4
Cough	1 (1.0)	1 (1.0)	0 (0)	3 (2.9)	1 (1.0)	0.5
Runny nose	3 (3.0)	1 (1.0)	1 (1.0)	0 (0)	2 (2.0)	0.3
Stuffy nose	4 (4.0)	5 (4.8)	6 (5.8)	5 (4.8)	2 (2.0)	0.7
Hot to the touch or has fever	4 (4.0)	1 (1.0)	2 (1.9)	2 (1.9)	2 (2.0)	0.7
Other signs of illness^*d*^	1 (1.0)	1 (1.0)	1 (1.0)	1 (1.0)	0 (0)	≥0.9
At least one of the symptoms listed above	20 (20)	23 (22)	29 (28)	20 (19)	18 (18)	0.4
More than one of the following: vomiting, diarrhea, and/or abdominal distension	0 (0)	0 (0)	0 (0)	0 (0)	0 (0)	NA
Study personnel-observed signs, *n* (%)^*e*^						
Number of visits at which an infant was examined	288	302	306	293	297	
Poor feeding (not sucking effectively)^*f*^	0 (0)	0 (0)	0 (0)	0 (0)	0 (0)	NA
Jaundice	9 (3.1)	8 (2.6)	13 (4.2)	8 (2.7)	5 (1.7)	0.8
Skin pustules or abscess	1 (0.3)	0 (0)	2 (0.7)	2 (0.7)	1 (0.3)	0.8
Skin rash	0 (0)	2 (0.7)	2 (0.7)	1 (0.3)	1 (0.3)	≥0.9
Sunken, red, oozing, or swollen eyes^*g*^	0 (0)	1 (0.3)	1 (0.3)	0 (0)	1 (0.3)	≥0.9
Severe lower chest wall in-drawing^*f*^	0 (0)	0 (0)	0 (0)	0 (0)	1 (0.3)	0.5
Fever (≥37.5°C)^*f*^	1 (0.3)	0 (0)	1 (0.3)	0 (0)	0 (0)	0.5
Hypothermia (<35.5°C)^*f*^	0 (0)	0 (0)	0 (0)	1 (0.3)	0 (0)	0.3
Convulsions^*f*^	0 (0)	0 (0)	0 (0)	0 (0)	0 (0)	NA
No movement, movement only with stimulation, or unconscious^*f*^	1 (0.3)	0 (0)	0 (0)	0 (0)	0 (0)	0.09
Other signs of illness^*h*^	2 (0.7)	2 (0.7)	1 (0.3)	0 (0)	1 (0.3)	0.6

^*a*^ From the day after the first IP dose up to and including 3 days after the last dose.

^*b*^ *P*-values were based on permutation testing (see text for details). NA indicates that statistical testing of comparisons was not performed due to zero events.

^*c*^ Count (*n*) and percentage of infants with a symptom(s) reported at least once among all infants who received at least one IP dose.

^*d*^ Other symptoms reported by caregivers included noisy breathing, projectile vomiting, poor feeding, fast or difficult breathing, and unusually sleepy or could not wake from sleep.

^*e*^ Count (*n*) and percentage of visits at which the sign was observed among all visits at which the relevant examination was conducted.

^*f*^ Sign of clinical severe infection.

^*g*^ *n*_Overall_ = 1,478; *n*_Placebo_ = 288; *n*_LP1_ = 300; *n*_LP1+FOS_ = 304; *n*_LP7_ = 292; *n*_LP7+FOS_ = 294; due to missing evaluations of sunken, red, oozing, or swollen eyes

^*h*^ Other signs of illness observed by study personnel included abdominal distension, mouth breathing, nasal discharge/rhinorrhea, umbilicus red, discolored, or discharging pus, and elevated respiratory rate.

Overall, the IP was well-tolerated and there were no differences in tolerability across intervention groups. Less than 1% of all attempts to administer IP resulted in an immediate intolerability event, including vomiting within 30 minutes of consuming the IP or spitting out the IP dose within 1 minute of IP administration, and there was no difference in immediate intolerability events between intervention groups. Few IP doses (between 0 and 0.4% across groups) were deferred or refused by caregivers; IP doses were refused due to concerns that the IP would not be tolerated (*n* = 2) and due to refusals from other family members (*n* = 2). Among infants who received at least one IP dose, 11 (2.1%) infants had their regimen temporarily discontinued, with no statistically significant difference across intervention groups. The proportion of infants who had their IP regimen permanently discontinued (*n* = 6, 1.2%) did not differ across groups (Table 5). There were no instances where the IP regimen was permanently discontinued due to intolerability or a clinical adverse event; reasons for permanently discontinuing the IP regimen included the deferral of three consecutive IP doses due to caregiver refusal (*n* = 4), a caregiver expressing an intent to continue refusing IP doses (*n* = 1), and an infant reaching 21 days postnatal age, the maximum age at which IP could be administered, before all seven IP doses were administered (*n* = 1).

**TABLE 5:** Tolerability of the investigational product by intervention group

Parameter	Intervention group					
	Placebo	LP1	LP1 + FOS	LP7	LP7 + FOS	*P^a^*
Outcomes by IP attempt, *N* total attempts	698	730	723	724	715	
IP attempts with an immediate intolerability event,^*b*^ *n* (%)	1 (0.1)	5 (0.7)	2 (0.3)	0 (0)	1 (0.1)	0.09
Vomiting within 30 minutes of IP, *n* (%)	1 (0.1)	4 (0.5)	2 (0.3)	0 (0)	1 (0.1)	0.3
Spitting^*c*^ out dose within 1 minute of IP, *n* (%)	0 (0)	1 (0.1)	0 (0)	0 (0)	0 (0)	0.8
Immediate intolerability event following IP attempts for dose 1,^*b*^^,*d*,*e*^ *n* (%)	0 (0)	1 (0.9)	1 (1.0)	0 (0)	0 (0)	>0.9
Immediate intolerability event following IP attempts for doses 2–7,^*b*^^,*f*^ *n* (%)	1 (0.2)	4 (0.6)	1 (0.2)	0 (0)	1 (0.2)	0.2
Outcomes by IP visit, *N* total visits	710	733	725	733	717	NA
Incomplete dose due to caregiver refusal or deferral,^*g*^ *n* (%)	2 (0.3)	2 (0.3)	2 (0.3)	3 (0.4)	0 (0)	0.7
Incomplete dose due to immediate post-ingestion intolerance, *n* (%)	0 (0)	0 (0)	0 (0)	0 (0)	0 (0)	NA
Outcomes by infant, *N* total participants^*h*^	101	105	103	104	102	NA
Infants with at least one dose refusal or deferral by caregiver,^*i*^^,*j*^ *n* (%)	2 (2.0)	2 (1.9)	2 (1.9)	3 (2.9)	0 (0)	NA
IP temporarily discontinued,^*i*^ *n* (%)	1 (1.0)	3 (2.9)	1 (1.0)	5 (4.8)	1 (1.0)	0.2
IP permanently discontinued, *n* (%)	2 (2.0)	3 (2.9)	0 (0)	1 (1.0)	0 (0)	0.3

^*a*^ *P*-values were derived from permutation testing (see text for details). Statistical testing of comparisons of incomplete IP dose administration across groups was only performed for outcomes where the IP administration visit was the unit of analysis and for events of temporary or permanent IP discontinuation. NA indicates cases where statistical testing of comparisons was not performed.

^*b*^ Directly observed by study personnel within 30 minutes of IP dose.

^*c*^ Study personnel observed for dripping/drooling or spitting out the IP dose, but no events of dripping/drooling were reported.

^*d*^ Dose 1 contained active IP in all groups except placebo.

^*e*^ Among all IP attempts for dose 1, *N*_Overall_ = 517; *N*_Placebo_ = 101; *N*_LP1_ = 106; *N*_LP1+FOS_ = 104; *N*_LP7_ = 104; *N*_LP7+FOS_ = 102.

^*f*^ Among all IP attempts for doses 2 to 7, *N*_Overall_ = 3073; *N*_Placebo_ = 597; *N*_LP1_ = 624; *N*_LP1+FOS_ = 619; *N*_LP7_ = 620; *N*_LP7+FOS_ = 613.

^*g*^ A deferral was counted if the infant was available for the visit but a caregiver was not available to consent to proceed with IP administration.

^*h*^ Among infants who received at least one dose.

^*i*^ Despite a break in IP administration, all infants received all seven doses. Infants who eventually had IP permanently discontinued were not included in the “IP temporarily discontinued” category.

^*j*^ Three infants with caregiver dose refusals are also included in the “IP temporarily discontinued” category because the study medical officer agreed that IP should be paused until further review.

Across all intervention group permutation tests of tolerability outcomes, adverse events, *ad hoc* medical assessments, and biochemistry and hematological outcomes (*n* = 218 tests), the proportions of *P*-values that were <0.05 or between 0.05 and <0.1 were 4.1% and 4.6%, respectively, consistent with the simulated type 1 error rate of the permutation test of 4.2%, thereby strongly suggesting that there were no true differences in these domains among intervention groups. By contrast, among all between-hospital enrollment site permutation tests of tolerability outcomes, adverse events, *ad hoc* medical assessments, and biochemistry and hematological outcomes (*n* = 196 tests), the proportions of *P*-values that were <0.05 or between 0.05 and <0.1 were 15.3% and 8.2%, respectively, suggesting a pattern of true differences in these outcomes between hospital enrollment sites.

## DISCUSSION

Based on a prior study (6) that showed that a 7-day course of LP202195 plus FOS resulted in sustained *L. plantarum* colonization of the infant GI tract for several months beyond the period of IP administration, we expected that short-duration LP202195 regimens, and possibly even a single dose, could lead to stable colonization of LP202195 beyond the IP administration period and until at least 2 months of age, thereby exerting potential probiotic/synbiotic effects during the age window with the highest risk of SI and mortality. We did not apply a formal definition of probiotic colonization in this study, particularly since detection of LP202195 in stool collected during or shortly after IP administration may reflect its passive passage through the intestinal tract (7). However, we considered that a steady-state elevated AA of LP202195 in stool samples collected after cessation of probiotic administration (i.e., 14–60 days of age) would reflect *in vivo* replication of the LP202195 strain and thereby serve as evidence of intestinal colonization. We found no such evidence of LP202195 colonization in Bangladeshi infants in this study; rather, the average AA of LP202195 declined during and after the period of IP administration. A minority and declining proportion of samples beyond 21 days since first dose had a high AA, such that the median AA was no different from the placebo group by 2 months of age. A lack of colonization in the post-intervention period has been commonly observed in trials of other probiotics (13–15), with some exceptions, including the report of long-term persistence of a *Bifidobacterium longum* subspecies *infantis* probiotic in infants in the United States (16). The prior LP202195 trial in a hospital in India was conducted in a small cohort (*n* = 31) of infants (>35 weeks gestation and >1,800 g at birth), all of whom were delivered via C-section (6). Unlike the present study in which we used molecular techniques to specifically identify and quantify the strain of *L. plantarum* that was administered to participants (12), the prior trial relied on conventional culture and microbiological techniques to assess the presence, but not the abundance, of *L. plantarum* in stool (6). The use of techniques with lower strain specificity may have resulted in artificially high probiotic detection rates. Differences in the enteric microbiota of the infants in each study may also have influenced the ability of LP202195 to colonize the infant GI tract, as has been demonstrated in other probiotic studies (7, 17). Colonization is not generally thought to be necessary for a probiotic to exert functional or clinical effects (18); however, very few studies have explored the effect of probiotic/synbiotic administration on probiotic colonization in infants (19) and none have explored the association between colonization and health outcomes. The absence of colonization in this population does not, on its own, imply that the synbiotic lacks clinical benefits, particularly as LP202195 could be administered as a daily or less frequent intermittent dose over a longer postnatal period that overlaps with the entire age window in which the clinical outcome is of most interest.

The absence of a significant effect of FOS on LP202195 AA with either the 1- or 7-day regimens suggested that FOS may not provide sufficient value to warrant the use of a FOS-containing synbiotic rather than probiotic alone. The genome of LP202195 contains a conserved operon involved in FOS metabolism (20), and it is well-documented that *L. plantarum* strains, including strain ATCC 202195 (20), have the potential to metabolize a wide array of carbohydrates in a strain-specific manner (21). However, an *in vitro* study that assessed the growth of 77 different strains of *L. plantarum* in the presence of diverse prebiotics, including FOS, revealed that only 1 of the 77 strains studied was able to grow to a high cellular density on FOS (21). While our results indicate that FOS had no effect on the post_14-60_ stool AA of LP202195, the role of FOS in influencing other microbes within the microbiota, thereby impacting the viability of LP202195, or in contributing in other ways to the clinical effect of the intervention on neonatal sepsis that was previously reported (5) should be explored in future work.

The reporting of adverse events (AEs) and serious adverse events (SAEs) in probiotic and/or synbiotic trials often lacks sufficient detail to draw conclusions regarding IP safety (22). Here, we implemented both an active and passive surveillance system to rigorously identify and document AEs and SAEs and found no evidence suggesting that LP202195 poses any safety risks to infants, consistent with prior reports on this synbiotic regimen (5). We found no differences across intervention groups in any of the tolerability or safety outcomes investigated, including biochemical and hematological analyte concentrations, which have not previously been investigated for this probiotic strain. The safety of LP202195 is further supported by prior reports that the genome contains no antimicrobial resistance genes or other concerning virulence factors, and that it is susceptible to several clinically important antibiotics (20). There were no cases of bacteremia associated with LP202195 identified in this or prior studies; however, if cases of probiotic-associated sepsis arise in the future, as has occurred for other probiotic regimens (23–28), it is reassuring that the strain is known to be susceptible to first-line antibiotic regimens and that there are validated molecular methods available to detect and quantify LP202195 with high specificity. Lastly, both probiotic safety, including the absence of undeclared microbial contaminants (29), and efficacy (30) can be impacted by manufacturing procedures. In the reports of two prior trials conducted in India (5, 6), limited detail was provided regarding the production of LP202195 + FOS, and we were unable to trace their original supply chain when designing this study in 2018. For the IP used in the present trial, we confirmed the strain’s identity (20), that manufacturing conditions (by International Flavors & Fragrances Inc. [IFF], formerly DuPont) were scalable and adhered to Good Manufacturing Practices using the identified strain, and that a cold chain was maintained until the IP was administered to participants.

This study was under-powered to precisely assess the effect of the intervention on SI, which was not the primary endpoint of this phase 2 trial, and the SI rates (8.9% in placebo group in India vs 5.8% in placebo group here) and culture-confirmed SI rates (1.2% in placebo group in India vs 0% in placebo group here) were lower in this setting than in the community-based trial in India (5). We were also under-powered to precisely assess the effect of the intervention on hospitalization events, which we would expect to see if there was a true effect on SIs. Thus, we are unable to confirm or refute the effect of the intervention on newborn sepsis that was previously reported.

Probiotic cross-contamination, whereby a probiotic organism is unintentionally transferred to humans or environmental surfaces, may mask the true efficacy of an intervention if a large proportion of infants in the control group are affected by cross-contamination. Compared to other trials, like the ProPrems (31, 32) and PiPS (33) trials, which reported that 7.9% and 37%, respectively, of infants who did not receive the probiotic had detectable probiotic in their stool, the cross-contamination rate in this trial was relatively low (4% of post_14-60_ stool samples collected in the placebo group contained detectable levels of LP202195). We previously demonstrated that LP202195 was undetectable in infant stool samples that were collected in the trial catchment area prior to introducing the probiotic (12), suggesting that contamination observed in the placebo group was unlikely due to the presence of naturally occurring, highly related strains of LP202195. Given the randomized design of the study, contamination was expected to be evenly distributed across intervention groups. Therefore, we believe it is unlikely that this low level of contamination impacted our findings.

Several limitations of this study should be acknowledged. First, we assumed that the presence and quantity of LP202195 in an infant stool sample was reflective of the presence and average abundance of LP202195 in the GI tract. However, emerging evidence suggests that probiotic detection in stool is not a reliable proxy for gut colonization (7, 34). The invasive collection of intraluminal samples was not ethical or feasible in this trial, and innovative methods to non-invasively collect samples from different regions of the GI tract (35) are not yet widely available or ready for use in infants. Given that our primary outcome window focused on a period that occurred after IP administration, and that a post-administration steady-state AA was not maintained in any of the probiotic or synbiotic groups, we believe there is sufficient evidence to support the conclusion that LP202195 did not colonize the gut in most infants. The decline in LP202195 median AA over several weeks was likely due to gradual shedding of probiotic bacterial DNA from the originally ingested bacteria rather than due to the detection of DNA from the progeny of the administered cells, yet techniques are not readily available to distinguish between administered and progeny probiotic cells. Since probiotic strain detection in stool samples during the administration period may reflect passive transit along the intestinal tract, we were unable to determine if LP202195 transiently colonized the infant gut, and it remains unknown whether a longer duration regimen (>7 days) of LP202195 would more effectively promote colonization in this population. Second, given a lack of empirical data at the time the trial was designed, the non-inferiority margin of −1 log cells/µg DNA was arbitrarily selected. Thus, our ability to make inferences using the pre-specified non-inferiority margin is limited, particularly given the lower than anticipated observed AAs of LP202195; the maximum stool AA of LP202195 was approximately 10^5^ cells/µg DNA, which is several orders of magnitude lower than what has been reported for other probiotics (16). However, the same analyses were leveraged to assess whether there were statistical differences in the mean AA in each active IP-containing group relative to the LP7 + FOS group, enabling conclusions to be drawn regarding the effect of FOS on post_14-60_ AA of LP202195. It is possible that antibiotic exposures in this population contributed to the lower-than-expected maximum stool AA of LP202195; however, given that the mean AA among stool samples collected prior to any antibiotic exposures was still relatively low, antibiotics alone cannot explain this observation and other factors are likely involved. Third, due to variations in the number of stool samples per infant for which qPCR was generated, we elected not to analyze the longitudinal dynamics of LP202195 AA in individual infants; however, even with higher numbers of samples per infant, it may be challenging to find meaningful between-infant comparisons (e.g., responders versus non-responders) given the substantial within-infant variability in AA. Fourth, while safety assessments were robust, we did not assess the effect of the intervention on metabolic or immune pathways, for which there are plausible mechanisms by which probiotic interventions can intervene (36). Fifth, there were unexpected differences in absolute stool abundance of LP202195 between samples collected at the two hospital enrollment sites. In *post hoc* analyses, we were unable to identify factors to explain these differences and speculate that they may be attributable to operational factors or differences in the stool microbiota or metabolome of infants at different sites. However, site was included as a fixed variable in all models to account for these differences, and site was not an important modifier of the probiotic or synbiotic effect. Effects of the intervention on the infant gut microbiome will be the focus of future studies using metagenomic sequencing. Finally, our conclusions regarding the colonization of LP202195 may not be generalizable to other populations, as colonization is influenced by factors that vary by setting, including, but not limited to, the microbiome (7).

In conclusion, a 1- or 7-day regimen of LP202195 with or without FOS did not result in intestinal colonization of LP202195, defined here as prolonged elevated AA of LP202195 in stool samples collected beyond the IP administration period, in infants born in two public hospitals in Dhaka, Bangladesh. This finding is inconsistent with a prior study of this synbiotic regimen in India. Therefore, additional trials are warranted to confirm the previously observed effects of LP202195 on infant clinical outcomes in the absence of LP202195 colonization, or should be designed to explore the effect of longer regimens of LP202195 (>7 days) which span the entire age window of the clinical outcome of interest, without FOS, and therefore not assuming that colonization is required to impart clinical efficacy.

## MATERIALS AND METHODS

### Study design and oversight

This study was a randomized, placebo-controlled, double-blinded, five-arm trial of neonatal oral administration of LP202195 (10^9^ CFU/day) with or without FOS. At enrollment, participants were randomly allocated to one of five intervention groups: placebo; 1-day regimen of LP202195 with or without FOS (LP1 + FOS or LP1, respectively); or 7-day regimen of LP202195 with or without FOS (LP7 + FOS or LP7, respectively). Enrollment took place between 7 January 2022 and 24 April 2022. Participant follow-up concluded on 13 November 2022, when the last participant exited the trial. The trial protocol was registered at ClinicalTrials.gov (NCT05180201). A data safety and monitoring board was appointed by the International Centre for Diarrhoeal Disease Research, Bangladesh (icddr,b), the lead implementation site, to review all SAEs. A trial steering committee, composed of all study co-investigators, oversaw the trial conduct, and RTI International externally monitored activities and adherence to protocols.

### Setting and participant eligibility

Infants aged 0 to 4 days of age were screened for eligibility to enter the study at two government hospitals in Dhaka City, Dhaka, Bangladesh: MCHTI and MFSTC. Infants were eligible for inclusion if they were delivered in either of the two study hospitals, were 4 days of age or younger (day of birth was defined as day 0), ≥1,500 g at birth, orally feeding at the time of eligibility assessment, and the caregiver intended to maintain residence within the defined catchment area until the infant was 60 days of age. Infants were not eligible to participate if their birth weight was <1,500 g, death or major surgery was considered highly probable during the first week of age, they had a major congenital anomaly of the gastrointestinal tract, they were receiving mechanical ventilation and/or cardiac support (e.g., inotropes) and/or administration/prescription of parenteral antibiotics at the time of eligibility assessment, there was evidence of maternal HIV infection or prior anti-retroviral treatment for presumed HIV infection, their mother had used, prenatally or in the postpartum period, a non-dietary probiotic supplement, they received postnatal administration of any non-dietary probiotic or prebiotic supplement, they were participating in another clinical trial at the time of eligibility assessment, they were residing in the same household as a non-twin infant aged <60 days who was previously enrolled in this trial or the other study that was implemented using the same research infrastructure (NCT04012190), or the infant was one of three or more liveborn infants from the same pregnancy. Parents or legal guardians of all infants provided written informed consent prior to enrollment by a study medical officer. Enrollment was stopped after 500 infants had completed the day 10 follow-up visit; a total of 519 participants were enrolled.

### Randomization and blinding

Participants were randomly assigned to one of the five intervention groups using a 1:1:1:1:1 allocation ratio according to a random sequence generated using permuted block randomization with a block size of 10, stratified by hospital enrollment site. Randomization was performed by RTI International, and allocation sequences were provided to IFF (www.iff.com), formerly DuPont, to enable packaging and labeling of IPs. Participants, investigators, and all study staff, including data analysts, were blinded to the intervention group allocation. The allocation list was maintained by RTI International until recruitment and data collection concluded. Intervention group labels were unmasked on an analysis-by-analysis basis and only after statistical analysis plans were approved by the trial’s lead statistician (E.P.), and findings were reviewed overall by a randomly assigned “dummy” group variable and by a masked, but true, intervention group variable.

### Interventions

Active (10^9^ CFUs of LP202195 with or without FOS) and placebo (maltodextrin) IPs were manufactured and packaged by IFF in Madison, WI, USA, in accordance with US Food & Drug Administration’s Good Manufacturing Practices. Prior work by our group demonstrated that the strain of LP202195 used in this trial was genetically identical to the strain of LP202195 that was used in the community-based trial conducted in rural India (20). Each daily dose was packaged into a 5 mL vial. Since the first dose in the LP1 and LP1 + FOS arms differed from subsequent doses, all first dose vials, irrespective of trial group, were marked with a red cap. Placebo group vials contained 250 mg of maltodextrin, while active IP vials contained 10^9^ CFU of LP202195, with or without 150 mg of FOS, and 100 mg or 250 mg of maltodextrin, respectively. All IPs and placebo resembled a fine, white powder and were identical in appearance, weight, scent, taste, and packaging. Boxes containing IP vials were stored at −20°C at icddr,b until they were transported to field sites for assignment to enrolled participants. During transit and at the field sites, IPs were always maintained between 2°C and 8°C. The temperature of IP-containing freezer and refrigerators was routinely monitored and recorded twice per day. Among IP vials that were remaining at trial endline, 30 vials were sent to IFF for analysis. Some vials were selected from boxes of IP that had never been assigned to a participant and thus remained in the freezer throughout the trial, whereas others were from boxes of IP that had been assigned to participants and were therefore handled in the field. Irrespective of storage and handling conditions, results indicated minimal to no loss of probiotic viability at trial endline.

### Administration of investigational products

Up to two IP vials, the assigned IP vial and the matching overage vial that was carried to account for possible handling errors or intolerability events, were transported to the point of IP administration (hospital or home) in temperature-controlled cold boxes. IPs were prepared at the point of administration and were always orally administered by study personnel. IPs were preferentially reconstituted in approximately 3 mL of human milk, although 3 mL of sterile water or a mixture of water and human milk could also be used. IP vials were shaken vigorously until the IP dissolved. Efforts were made to administer IPs once per day for 7 consecutive days, up to a maximum of seven doses per infant. IP administration was preferentially timed to occur prior to or at the beginning of an infant feed and infants were observed for 30 minutes after receiving IP. If any sign of intolerance was observed, a repeat dose was attempted if permitted by the caregiver, up to a maximum of two dose attempts per visit. For the first dose only, the second attempt to administer IP could take place on a later date, if necessary. The final IP dose could be administered up to and including day 21 postnatal age.

### Surveillance and data collection

Participants were followed from enrollment until 6 months (180 days) of age; however, the 6-month visit could have been completed up to 9 months of age. Routine clinical assessments were conducted by study personnel on the day of enrollment, 3 days post enrollment, 6 days post enrollment, and on days 10, 14, 21, 28, 35, 42, 49, 56, 60, 90, and 180 postnatal age, with allowances for rescheduling. If in-person visits were not feasible, self-reported data were collected by telephone. At each routine clinical assessment visit, study personnel documented caregiver-reported concerns about the infant’s health based on a standardized list of symptoms. Caregiver-reported symptoms prompted a clinical evaluation of the infant where the presence or absence of numerous clinical features, including signs of possible clinical severe infection (CSI), were recorded. Signs of CSI included poor feeding (not sucking effectively or not sucking at all, based on direction observation by a study medical officer [SMO]); lethargy (movement only when stimulated or not moving at all, based on direct observation by an SMO); convulsions, based on direct observation by an SMO or strongly suspected by a study medical officer based on caregiver or community health research worker (CHRW) report; severe chest in-drawing based on direct observation by an SMO; fever (axillary temperature ≥37.5°C or rectal temperature ≥38°C); and hypothermia (axillary temperature <35.5°C or rectal temperature <36°C). CSI signs prompted same-day referral to the nearest study hospital for assessment. Caregivers were encouraged to contact study personnel or present to a study hospital if any concerns about the infant’s health arose between scheduled visits. Infants underwent active and passive surveillance for possible CSI from enrollment until 60 days of age. Passive surveillance for infant illness, hospitalization, and other SAEs continued until 180 days of age. In the event of infant death, a verbal autopsy was conducted 7 to 15 days after the event, to determine the cause of death (37). Except for laboratory results, data were recorded using a tablet-based data capture system.

### Specimen collection and processing

Infant stool samples were routinely collected at enrollment, on days 4, 5 or 7 (randomly determined), 10, 14, 21, 28, 42, 60, 90, and 180 postnatal age, with allowances for rescheduling. Except for enrollment samples, most infant stool specimens were collected in the participant’s home by trained study personnel from a plastic wrap-lined diaper (≤1 week of age) or sanitized plastic sheet (>1 week of age). Samples were homogenized, aliquoted, and placed in the vapor phase of liquid nitrogen within 20 minutes of defecation. Aliquots were stored at −70°C or colder until analyzed.

Phlebotomists collected a single routine venous blood sample (~3.5 mL) from infants on either day 9 or day 60 (randomly determined) using standard blood collection procedures and allowing for sample collection visits to be rescheduled. Routine blood was divided into a serum vacutainer tube (BD, Franklin Lakes, NJ, USA, Mfr. No. 366668) (~2 mL) and EDTA microtainers (BD, Franklin Lakes, NJ, USA, Mfr. No. 365975) (~0.5 mL each). Serum vacutainers were mixed by five gentle inversions, left to clot at room temperature for 30 minutes, and then centrifuged at 2,000 × *g* for 10 minutes at room temperature. Serum was aseptically transferred to a foil-wrapped tube and stored at 2°C–8°C until analyzed. EDTA microtainers were mixed by 8–10 gentle inversions and one aliquot was stored at 2°C–8°C until analyzed. Samples were transported to clinical testing labs no later than 10 hours after collection.

The collection of non-routine blood and urine samples was attempted from infants who had any sign of CSI confirmed by a study medical officer or if a non-study treating physician recommended a septic work-up. Septic work-up blood samples were collected and processed as described for routine blood samples except that the target total volume collected was approximately 5 mL, and the first 1 mL of blood was prioritized for blood culture purposes. Urine samples were collected following standard “bagged” or “clean-catch” protocols. The collection of non-routine cerebrospinal fluid (CSF) samples was also standardized; however, no CSF samples were collected.

### Stool sample selection and laboratory analysis

A random sub-set of routinely collected infant stool samples were analyzed by qPCR. Approximately 90% of the analyzed samples were selected using a probability-based algorithm. The first sample from all infants was always selected and remaining samples were selected on a rolling basis using a random Bernoulli distribution with a fixed selection probability. Two additional rounds of random sample selection were implemented to increase sample sizes across the age range, and to ensure that at least one sample within the primary microbiological outcome window (14 to 60 days, inclusive) had been selected from all infants, where possible.

qPCR assays were conducted using methods that have been previously described (12). In brief, standard curves were prepared from DNA isolated from quantified cultures of LP202195. Total DNA was extracted from approximately 150 to 200 mg of stool using the QIAamp fast DNA stool mini kit (QIAGEN, Germany, Catalog # 51604) with a bead beating step to facilitate cell disruption (12), and using a final eluant volume of 200 µL. qPCR assays were performed using primers (LP202195-Forward:5´5´GAG CAG ATT ATT GGC GAT TGG-3´; LP202195-Reverse:5´5´CAT CTA GCG ATA ACG TTC CTT G-3´MGB-NFQ) and a probe (LP202195-Probe; 5´6-FAMTM reporter/GCC TTT GTA TAC GAC GTT CAT TTA GCT AGT C/MGB-NFQ) that were previously designed to detect and quantify LP202195 (12). Cell counts (without any normalization step) were estimated from the qPCR standard curve and the measured cycle quantification (Cq) value. For samples that amplified with a Cq value between the assay’s lower limit of detection (LLOD) and lower limit of quantification (LLOQ), cell counts were extrapolated from the standard curve. If Cq values were below the LLOD, cell counts were imputed at one-half of the LLOD in primary analyses. The LLOD and LLOQ for the assay were previously determined to be 15 and 150 cells per reaction, respectively, without any normalization to DNA or stool quantity (12). To assess assay replicability, two independent qPCR plates were run using a single DNA aliquot from 282 stool samples collected during the trial. Of the 282 samples run in duplicate, 86 had measured (non-imputed) cell counts in both runs; the median inter-assay coefficient of variation of measured log_10_ cell counts was 3.7% with an inter-quartile range of 1.9% to 5.9%. There was a strong linear relationship between measured cell counts (Pearson correlation coefficient = 0.97; *n* = 86), and high between-run agreement on classification as having measured vs imputed cell counts (96%, Kappa = 0.92; *n* = 282). Absolute abundance, expressed in units of cells per µg DNA (in primary analyses) or cells per g stool (in sensitivity analyses), was calculated using the following equations (*C*)/(*V*_DNA_Reaction_ × *D*) or (*C* × *V*_DNA_Eluant_)/(*V*_DNA_Reaction_ × *M*), respectively, where *C* was cell counts obtained from the standard curve, *V*_DNA_Reaction_ was the volume of unknown DNA template added to the reaction well, *D* was the DNA concentration measured via nanodrop, *V*_DNA_Eluant_ was the total volume of DNA eluant obtained during extraction, and *M* was the measured mass of the stool sample aliquot used for extraction.

Routine hematological (complete blood count with differential) and biochemical analyses (procalcitonin, high-sensitivity C-reactive protein, creatinine, alanine transaminase, glucose, albumin, total, direct, and indirect bilirubin) on plasma and serum, respectively, were measured at icddr,busing standard clinical lab protocols. Among septic work-up blood samples, ~1 mL of blood was injected into a BD BACTEC Peds Plus/F Culture Vial, which was stored at room temperature until it was cultured in a BACTEC system at the Child Health Research Foundation. Beep-positive samples were sub-cultured and Gram-stained. Urine samples were evaluated using standard dipstick procedures and cultured on selective media to identify pathogens.

### Outcomes

The primary outcome was the stool AA of LP202195 from days 14 to 60 postnatal age, inclusive, based on a maximum of five post-intervention period stool samples collected per infant during the period. In primary analyses, the AA of LP202195 was defined as the log_10_ number of cells of LP202195 per mass (µg) of extracted DNA. Infant stool samples were also categorized as having a “high” vs “low” AA of LP202195 using an empirically determined threshold of 3 log_10_ cells/ µg DNA; the threshold was equivalent to the mean minus 1 standard deviation among samples with a Cq value above the LLOD of the qPCR assay. There is no standard definition of probiotic colonization, but in the interpretation of the AA outcomes, we qualitatively considered that steady-state persistence of a significantly elevated average AA with a majority of samples having high AA beyond the period of IP administration, and for at least 2 months (i.e., throughout the 14- to 60-day post-intervention period), would provide evidence of intestinal colonization.

Tolerability outcomes included immediate post-ingestion intolerability signs, incomplete IP dose administration events, dose refusals or deferrals by a caregiver, and events of permanent or temporary IP discontinuation. Immediate post-ingestion intolerability was assessed by directly observing infants for 30 minutes after IP ingestion for one or more of the following signs: dripping/drooling from the infant’s mouth or spitting out the dose within the first minute of IP administration; or vomiting within 30 minutes of IP administration. Doses were deemed incomplete if the IP dose could not be administered on the scheduled date due to intolerance or an AE. An IP dose was only considered refused or deferred if, at any point following the dose refusal or deferral, an IP dose was eventually administered; if a subsequent dose was never administered, the event was instead categorized as a permanent discontinuation. IP regimens were defined as temporarily discontinued based on study physician recommendation to pause the regimen, and only if the IP regimen was restarted. IP regimens were deemed permanently discontinued if there was concern that an AE was related to IP administration or an adverse reaction to the IP, f three consecutive IP doses were deferred due to caregiver refusal and there was no evidence of a clinical reason for deferral, or if the caregiver expressed an intent to discontinue IP or refuse all subsequent doses.

Clinical AEs, including both caregiver-reported symptoms and study personnel observed signs, were documented at scheduled and unscheduled visits conducted by CHRWs and at unscheduled outpatient events with SMOs, both during and after the IP administration period. Caregivers reported symptoms to CHRWs at each routine clinical visit, before each IP administration event, during *ad hoc* phone calls and follow-up calls after the last IP dose, and during routine specimen collection visits that did not coincide with routine clinical visits. Reports were based on caregivers indicating the presence or absence of a given symptom from a comprehensive list of possible symptoms. Caregiver-reported symptoms during the IP period, defined as the day after the first IP dose, up to and including 3 days after the last IP dose, were presented as the number and proportion of infants who had at least one symptom during the period of interest among all infants who received at least one dose of IP. Caregiver-reported symptoms in the post-IP period were reported as the number of calendar days on which the caregiver reported the infant had symptoms within the specified time windows (3 days after last IP dose up to 60 days, and greater than 60 days to 180 days postnatal age). A standardized list of CHRW-observed signs was assessed at routine clinical visits, immediately prior to administering an IP dose if an examination was prompted by a caregiver-reported symptom, and at assessments prompted by *ad hoc* reports of infant illness or at follow-up visits. CHRW-observed signs, both during and after the IP period, were reported as the frequency of events, as a proportion of all visits at which the infant was examined, during the period of interest (during IP period, 3 days after last IP dose up to 60 days, and greater than 60 days to 180 days postnatal age). SMOs conducted clinical assessments and/or documented caregiver-reported signs during a scheduled baseline assessment at enrollment and during other *ad hoc* interactions with participants, both in person and over the phone, throughout the participant’s follow-up period. As above, standardized lists of possible signs and symptoms were used during SMO assessments. Signs and symptoms that were documented in the context of an inpatient hospitalization event were addressed as SAEs.

The primary safety outcome was SI for which *Lactobacillus* was deemed to be the causative agent based on its isolation from the blood or urine of an infant with SI. Secondary safety outcomes included SAEs such as infant death, hospitalizations, and SIs for which *Lactobacillus* was not deemed to be the causative agent. Non-injury deaths included all deaths that were not directly caused by physical trauma, as determined using a death certificate and/or review of verbal autopsy data and SAE reports by two physicians in Bangladesh. Hospitalizations were defined as any inpatient admission for acute illness that occurred from enrollment until 180 days. Hospitalizations associated with GI illness were a subset of all hospitalizations for which at any point during the hospitalization the infant had signs or symptoms associated with a GI illness or was diagnosed with a GI illness by a treating physician. Time at risk for hospitalizations and hospitalizations associated with GI illness was calculated as the time from enrollment to the day the infant exited the study (i.e., death or study completion) minus the duration of the hospitalization or hospitalization associated with GI illness, respectively. SI events were defined on the basis of at least one physician-documented sign of CSI and/or a diagnosis of sepsis or serious bacterial infection (SBI) and at least one of the following two criteria: physician decision to admit to hospital, administration of at least one dose of a parenteral antibiotic on the day when CSI/sepsis/SBI was first ascertained, and treatment, or physician intention to treat, with parenteral antibiotics for at least 5 consecutive days; or infant’s blood culture was positive for a pathogenic bacterial or fungal organism. An initial list of blood pathogens and contaminants was developed *a priori* and was later refined using clinical data and the list of organisms that were isolated from blood during the trial. Episode duration was defined from date of SI onset until the 3rd day after the last dose of antibiotics or hospital discharge, whichever was later. If SI criteria were met based on a physician’s intention to treat with antibiotics for at least 5 days and the infant was not hospitalized, then the episode was assumed to end on the 8th day after SI onset. Time at risk for SI was calculated as the time from enrollment until 60 days of age or participant exit or death, if earlier than 60 days, minus the duration of any SI episode(s). A combined outcome of SIs and non-injury deaths up to and including 60 days of age was also considered. Biochemistry and hematological markers of hematopoiesis, inflammation, renal function, and hepatobiliary status and function were measured and categorized, using age-specific standardized reference ranges that were adapted from the SickKids lab services guide and the CALIPER cohort database (38), based on whether analyte concentrations fell outside reference ranges.

### Sample size

Sample size calculations assumed that a detectable difference in the AA of *L. plantarum* in stool samples collected in the post-intervention period between days 14 and 60, inclusive, between LP202195-containing interventions versus placebo could be estimated with >99% power if 100 infants per trial arm were enrolled with five stool samples per infant, a within-infant intraclass correlation coefficient (ICC) of 0.25, a significance level of 5%, and allowing for 2% of stool samples in the placebo group to have detectable LP202195 and 17% of the stool samples in the LP202195-containing groups to have no detectable LP202195.

### Statistical analysis

Primary analyses were conducted using a complete-case intention-to-treat approach. Linear regression was used to model stool AA as a function of intervention group, adjusting for enrollment hospital, and using generalized estimating equations with an exchangeable correlation structure and robust standard errors to account for clustering within infants. Effect estimates were presented as mean differences with non-parametric bootstrapped 95% CIs. An intervention was deemed superior to the placebo group if the mean difference versus placebo was positive and the 95% CI for the between-group difference excluded zero. An intervention was deemed non-inferior to the LP7 + FOS group if the lower bound of the 95% CI for the between-group difference was not lower than the pre-specified non-inferiority margin of −1 log cells/µg DNA. Pre-specified sensitivity analyses were also conducted that varied the derivation of the AA outcome definition such that AA was expressed in units of cells/g stool or using only cell counts, without any normalization to mass of stool or DNA. Moreover, an alternate imputation method was used for determining cell counts in cases where the Cq value was less than the LLOQ, but greater than the assay’s LLOD, whereby cell counts were imputed as half the LLOQ, rather than extrapolating the cell count from the standard curve, as was done in primary analyses. Supplementary analyses using a per-protocol approach were restricted to infants who met the following pre-specified adherence criteria: received at least five of seven doses of IP by day 10 after birth and did not receive other probiotic interventions during the study period; received all seven doses of IP by day 10 after birth and did not receive other probiotic interventions during the study period; received their first dose of IP within 1 day of enrollment; received their first dose of IP by day 2 after birth; and had a range of 6 days between their first and last dose of IP, and received all seven doses of IP by day 10 after birth. Further pre-specified analyses explored the effect of the intervention on AA at varied age ranges and altered sample collection time points relative to receiving IP, as well as in sub-groups based on hospital enrollment site, mode of delivery, cumulative infant feeding pattern up to the time of stool sample collection, systemic antibiotic exposure prior to stool sample collection, and a dichotomized (high vs low) AA outcome variable. A *post hoc* analysis explored the effect of the intervention on the AA of LP202195 in sub-groups based on mode of delivery using only stool samples that were collected during the period of IP administration.

The effect of the intervention on temporal trajectories of median AA was modeled as a function of days since first dose using quantile regression with bootstrapped clustered standard errors to account for within-infant correlation, where AA was treated as a continuous dependent variable, and intervention group, days since first dose, interaction terms between each intervention group and age, and hospital enrollment site were included as independent variables in the model. Days since first dose was incorporated using linear splines to accommodate non-linearity in the association between time after first dose and AA, with knots at days 1, 2, 7, 14, and 60. Differences in median AA across intervention groups were expressed using marginal effects at the following discrete time points: 8, 14, 21, 28, 60, 90, and 180 days since first dose. To determine the number of days since first dose at which the AAs in each intervention group were no longer statistically different from placebo, marginal effects were also explored between days 28 and 60. The effect of the intervention on the predicted probability of having a high vs low AA of LP202195 by time since first dose was modeled using a mixed-effects logit model, adjusting for hospital enrollment site, and using linear splines for days since first dose, with knots at 2, 7, and 14 days since first does. All stool samples with qPCR data were included when modeling the temporal trajectories of AA. The ICC was calculated using a mixed-effects model of the log AA (in µg/DNA), adjusting for hospital site, intervention group, and days since first dose with the same knots as the quantile regression model described above.

Tests for differences in adherence across intervention groups or study sites was conducted using analysis of variance for normally distributed continuous variables, a Kruskal-Wallis test for non-normally distributed continuous/ordinal variables, or χ^2^ or Fisher’s exact tests (for small cell sizes) for categorical variables. Except for the test for differences in baseline characteristics across study sites, all assessments of between enrollment hospital differences are presented in Borealis, The Canadian Dataverse Repository (DOI: 10.5683/SP3/3QNINX). A regression-based approach, with adjustment for enrollment hospital, was conducted as a *post hoc* analysis of adherence across intervention groups. Permutation testing of sum of square differences with 1,000 to 10,000 iterations was used as a global test for significant differences between any two groups for tolerability outcomes, clinical adverse events during and after the IP period, *ad hoc* medical assessments of non-hospitalized infants, and the proportion of infants with biochemistry or hematology analyte concentrations outside of the reference range, accommodating for multiple outcomes within infants, where necessary, and adjusting for enrollment hospital. The expected type 1 error rate of the permutation test was determined using simulated data generated using a Bernoulli distribution, and a 5% event rate with 10,000 iterations; the proportion of null contrasts that led to a *P*-value less than 0.05 was 4.2%. Across all between-intervention group and between-hospital enrollment site tests in which the permutation test was applied, the total number and proportion of *P*-values that were less than 0.05 or between 0.05 and <0.1 was determined as means of globally assessing statistical significance. To address the multiplicity of comparisons, *P*-values were subjected to the Holm procedure for outcome “sets” defined based on outcome type (e.g., caregiver-reported symptoms) and, where applicable, the period in which the outcome was assessed (e.g., during the IP period), using an overall alpha of 0.05. The incidence rate of hospitalization and SI events were calculated using the total number of events and the total number of infant days at risk of a new event contributed by all infants, during the first 180 days (hospitalization outcomes) and first 60 days (SIs), respectively. Hospitalization and SI incidence rates and 95% CIs were estimated using intercept-only general linear models with a log link (Poisson family), and a logarithmic count of days at risk as the offset. The effect of the intervention on the rate of hospitalization (from enrollment to 180 days) or rate of SI (from enrollment to 60 days) in each active IP-containing group, relative to placebo, was estimated using Cox proportional hazard models with an Andersen-Gill extension to accommodate multiple episodes per infant, robust standard errors to account for clustering within infants, and adjusting for hospital enrollment site. The Holm procedure was used to account for multiplicity of testing. Enrollment hospital effect was estimated for the site covariate in the same model. Differences in average biochemistry analyte concentrations and hematological parameters across intervention groups, adjusted for enrollment hospital, and between enrollment hospitals were estimated using linear regression models. Highly skewed analyte concentrations and cell counts were log-transformed prior to modeling. A Wald test was used as a global test of any significant differences between any IP group and placebo for each analyte, and the Wald test *P*-value was presented. Wald test *P*-values for an outcome set defined by analyte type (biochemistry or hematology) and time interval (9 or 60 days), were subjected to the Holm procedure. The target alpha cutoff for the correction for each outcome set was 0.05.

All analyses were verified on a result-by-result basis through independent code review or by having a second data analyst rewrite derivation and analytical code (clinical adverse events during and after the IP period analyses, severe infection variable derivation, person-time calculations, and analyses, hospitalization analyses, merging qPCR, DNA extraction, participant ID, and stool collection date data, and analyses of absolute abundance relative to placebo or LP7 + FOS), followed by comparison and reconciliation, if and where needed. All analyses were performed using Stata version 16.1 (College Station, TX: StataCorp LLC.), and R statistical software (versions 4.2.3 and 4.4.1 [39]).

## Data Availability

De-identified data, code files, and all additional information required to reanalyze the data presented in this paper have been deposited at Borealis, The Canadian Dataverse Repository, and are publicly available. Digital object identifier (DOIs) for de-identified data, code files, and analyses by hospital enrollment site (10.5683/SP3/3QNINX), standardized operating procedures (10.5683/SP3/WKDQYY), and statistical analysis plans (10.5683/SP3/WKDQYY) are shown in parentheses.

## SUPPLEMENTAL MATERIAL

Supplemental Material msphere.01032-24-s0001.pdf Supplemental tables and figures.

ASM does not own the copyrights to Supplemental Material that may be linked to, or accessed through, an article. The authors have granted ASM a non-exclusive, world-wide license to publish the Supplemental Material files. Please contact the corresponding author directly for reuse.
