# Supplementary material for: Neonatal administration of Lactiplantibacillus plantarum ATCC 202195 with or without fructooligosaccharide in Bangladesh: a placebo-controlled randomized trial
Source: mSphere. 2025 Feb 24;10(3):e01032-24. doi: 10.1128/msphere.01032-24 (PMC11934310; doi:10.1128/msphere.01032-24)
Supplement: Supplemental Material — Supplemental tables and figures. [file msphere.01032-24-s0001.pdf]

Supplemental information associated with:

"Neonatal administration of *Lactiplantibacillus plantarum* ATCC 202195 with or without fructooligosaccharide in Bangladesh: a placebo-controlled randomized trial"

Lisa G. Pell[a], Huma Qamar[a], Diego G. Bassani[a,b,c], Cole Heasley[a], Celine Funk[a], Chun-Yuan Chen[a], Jakaria Shawon[d]*, Karen M. O'Callaghan[e], Eleanor Pullenayegum[b], Davidson H. Hamer[f], Rashidul Haque[d], Mamun Kabir[d], Tahmeed Ahmed[d], Ciobha O'Kelly[a,g], Md Iqbal Hossain[d], Afreen Z. Khan[a], Miranda G. Loutet[a], Mohammad Shahidul Islam[h], Shaun K. Morris[a,b,g], Prakesh S. Shah[g,i], Philip M. Sherman[g,j], Shamima Sultana[d], Abdullah Al Mahmud[d]*, Samir K. Saha[h], Shafiqul A. Sarker[d], Daniel E. Roth[a,b,c,g]#

[a]Centre for Global Child Health, Hospital for Sick Children, Toronto, Ontario, Canada

[b]Child Health Evaluative Sciences, Hospital for Sick Children, Toronto, Ontario, Canada

[c]Dalla Lana School of Public Health, University of Toronto, Toronto, Ontario, Canada

[d]Nutrition Research Division, International Centre for Diarrhoeal Disease Research, Bangladesh, Dhaka, Bangladesh

[e]Department of Nutritional Sciences, King's College London, London, United Kingdom

[f]Department of Global Health, Boston University School of Public Health and Chobanian & Avedisian School of Medicine, Boston, MA, USA

[g]Department of Paediatrics, Faculty of Medicine, University of Toronto, Toronto, Ontario, Canada

[h]Child Health Research Foundation, Dhaka, Bangladesh

[i]Department of Pediatrics, Mt. Sinai Hospital, Toronto, Ontario, Canada

[j]Cell Biology Program, Research Institute, Hospital for Sick Children, Toronto, Ontario, Canada

Running head: Randomized trial of *L. plantarum* in Bangladesh

#Address correspondence to Daniel E. Roth, daniel.roth@sickkids.ca

*Present address:

Jakaria Shawon, Department of Oncological Sciences, University of Utah, Salt Lake City, UT, USA and Huntsman Cancer Institute, Salt Lake City, UT, USA
Abdullah Al Mahmud, Shield Pharmaceuticals Corp. Ronkonkoma, NY, USA

Supplementary Information - Table of contents

Table S1. Maternal, household, delivery, and infant characteristics by enrolment hospital.

Table S2. Adverse events reported or observed during baseline medical assessments of infants by intervention group.

Table S3. Adherence to the investigational product (IP) by intervention group, with and without adjustment for enrolment hospital.

Table S4. Investigational product (IP) preparation and administration characteristics by intervention group.

Table S5. Stool samples collected and analyzed by qPCR by intervention group.

Table S6. Effect of administration of LP202195 with or without FOS on the absolute abundance of LP202195, relative to placebo or LP7+FOS, using alternative derivations of absolute abundance, alternative timings of sample collection, and per-protocol populations. Absolute abundance in log_{10} cells/µg DNA, except where noted.

Table S7. Effect of administration of LP202195 with or without FOS on the absolute abundance of LP202195 in stool samples, relative to placebo, by sub-groups. Analyses were conducted using $post_{14-60}$ samples, except where specified.

Table S8. Effect of administration of LP202195 with or without FOS on the absolute abundance of LP202195 in stool samples, relative to LP7+FOS, by sub-groups. Analyses were conducted using $post_{14-60}$ stool samples, except where specified.

Table S9. Absolute abundance of LP202195 at discrete time points following the first dose of investigational product in log cells/µg DNA and log cells/g stool by intervention group

Table S10. Routine biochemistry test results by intervention group.

Table S11. Routine hematology test results by intervention group.

Table S12. Adverse events in the post-investigational product (IP) administration period by intervention group.

Table S13. Adverse events reported or observed during ad hoc medical assessments of non-hospitalized infants in the period beyond the baseline assessment and up to 6 months by intervention group.

Table S14. Routine biochemistry and hematology testing sample sizes by analyte, timepoint, and intervention group.

Figure S1. Distribution, by intervention group, of amplification status of infant stool samples collected post-intervention (days 14 to 60) using a qPCR assay that was optimized for detection of LP202195.

Figure S2. *L. plantarum* ATCC 202195 (LP202195), when administered to neonates in Dhaka, Bangladesh for 1 or 7 days, with or without fructooligosaccharide (FOS), does not result in LP202195 colonization.

Table S1. Maternal, household, delivery, and infant characteristics by enrolment hospital.

	Enrolment hospital		
	MCHTI	MFSTC	p[a]
**Participants, N**	**132**	**387**	
Maternal age (years), median (25th, 75th)	22.5 (20, 26.5)	23 (20, 27)	0.37
Maternal education, n (%)			0.06
Little to no schooling[b]	29 (22)	127 (33)	
Secondary incomplete	45 (34)	110 (28)	
Secondary complete or higher	58 (44)	150 (39)	
Gravidity, median (25th, 75th)	2 (1, 3)	2 (1, 2)	0.30
First pregnancy, n (%)	47 (36)	149 (39)	
First live birth, n (%)	59 (45)	178 (46)	
Gestational age at delivery (weeks)[c], median (25th, 75th)	39.1 (38.3, 40.1)	39.0 (38.0, 40.0)	0.28
Term (≥37 weeks), n (%)	129 (98)	341 (90)	
Preterm (<37 weeks), n (%)	3 (2.3)	36 (9.5)	
Mode of delivery, n (%)			<0.001
Vaginal	52 (39)	225 (58)	
C-section	80 (61)	162 (42)	
Maternal peripartum antibiotics administered[d], n (%)	132 (100)	373 (96)	0.03
Asset index quintile[e], n (%)			0.007
1 (lowest)	16 (12)	93 (24)	
2	22 (17)	81 (21)	
3	33 (25)	84 (22)	
4	35 (27)	62 (16)	
5 (highest)	26 (20)	67 (17)	
Infant age at enrolment (days), median (min, max)	2 (0, 4)	1 (0, 4)	<0.001
Sex, n (%)			0.65
Male	69 (52)	211 (55)	

Female	63 (48)	176 (45)	
Birth weight (g), mean (SD)	2905 (323)	2883 (377)	0.55
Feeding pattern at or near enrolment[f], n (%)			0.86
Exclusively breastfed	114 (86)	331 (86)	
Not exclusively breastfed or not breastfed	18 (14)	55 (14)	

[a]p-value from t-tests or Mann-Whitney U Tests for continuous variables and Chi-square or Fischer's exact tests for categorical variables.

[b]Includes women with no education, and incomplete and completed primary school.

[c]Gestational age missing for 10 infants; 0 at MCHTI and 10 at MFSTC.

[d]Peripartum period refers to antibiotics that were administered in hospital during labour and/or in the operating theatre and/or after delivery, up to and including 4 days postpartum.

[e]Asset index scores and quintiles were generated using principal components analysis for all participants enrolled in the trial (n=519) and in a concurrently running observational study (n=1886) at the same study sites with the same eligibility criteria. Scores represent a summary measure of household wealth based on ownership of the following items: electricity, fan, mobile, almirah, fridge, television, chair, table, watch, bicycle, computer, freezer, pump, vehicle, rickshaw, phone, radio, autobike, cats, birds, poultry, dogs, goats, cows, and other animals. Lower scores reflect ownership of fewer items (i.e., lower wealth) and higher scores reflect the ownership of more items (i.e., higher wealth).

[f]Feeding pattern was derived using data collected at the first routine clinical visit after the first investigational product (IP) dose, or at the earliest available routine clinical visit if the infant did not receive IP. Otherwise, feeding pattern was derived using data collected at the baseline visit. Prelacteal feeds were not considered when ascribing feeding patterns. Feeding data were missing for 1 infant at MFSTC.

Table S2. Adverse events reported or observed during baseline medical assessments of infants by intervention group.

	Intervention group					
	Placebo	LP1	LP1+FOS	LP7	LP7+FOS	*p*[b]
**Participants, N[a]**	**104**	**105**	**103**	**104**	**103**	
**Caregiver-reported symptoms, n[c]**						
≥ 6 hours since last passed urine	0 (0)	1 (1.0)	0 (0)	0 (0)	0 (0)	0.8
Yellowing of skin or eyes	0 (0)	1 (1.0)	1 (1.0)	0 (0)	0 (0)	0.6
**Study medical officer-observed signs, n (%)[d]**						
Poor feeding (not sucking effectively)[e]	0 (0)	0 (0)	0 (0)	0 (0)	0 (0)	-
Jaundice[f]	0 (0)	1 (1.0)	1 (1.0)	0 (0)	1 (1.0)	0.6
Skin rash[g]	0 (0)	0 (0)	0 (0)	0 (0)	1 (1)	0.2
Sunken, red, oozing, or swollen eyes[h]	0 (0)	0 (0)	1 (1.0)	0 (0)	0 (0)	0.4
Elevated respiratory rate (≥60 breaths/min)	1 (1.0)	1 (1.0)	0 (0)	0 (0)	0 (0)	0.8
Severe lower chest wall indrawing[i,e]	0 (0)	0 (0)	0 (0)	0 (0)	0 (0)	-
Fever (≥37.5°C)[e]	0 (0)	0 (0)	1 (1.0)	0 (0)	0 (0)	0.4
Hypothermia (<35.5°C)[e]	0 (0)	0 (0)	0 (0)	0 (0)	0 (0)	-
Convulsions[e]	0 (0)	0 (0)	0 (0)	0 (0)	0 (0)	-
No movement, movement only with stimulation, or unconscious[e]	0 (0)	0 (0)	0 (0)	0 (0)	0 (0)	-

[a]All infants, irrespective of whether they received any doses of investigational product. The overall median age (25th, 75th) at the scheduled baseline medical assessment was 1 day (0 days, 2 days).

[b]p-values for across-group differences were based on permutation testing (see text for details).

[c]Count (n) and percentage (%) of infants with a symptom(s) reported at least once among all infants, whether or not any IP dose was received.

[d]Count (n) and percentage (%) of visits at which the sign was observed among all visits at which the relevant examination was conducted. All infants had one baseline visit examination, and thus the total number of visits at which an infant was examined, per group, was equal to the total number of participants, per group.

[e]Sign of clinical severe infection.

[f]$n_{Overall}$=517; $n_{placebo}$=104; n_{LP1}=104; $n_{LP1+FOS}$=103; n_{LP7}=103; $n_{LP7+FOS}$=103; due to missing evaluations of jaundice.

[g]$n_{Overall}$=518; $n_{placebo}$=104; n_{LP1}=105; $n_{LP1+FOS}$=102; n_{LP7}=104; $n_{LP7+FOS}$=103; due to missing evaluations of skin rash.

[h]$n_{Overall}$=518; $n_{placebo}$=104; n_{LP1}=104; $n_{LP1+FOS}$=103; n_{LP7}=104; $n_{LP7+FOS}$=103; due to missing evaluations of sunken, red, oozing, or swollen eyes.

[i]$n_{Overall}$=518; $n_{placebo}$=104; n_{LP1}=105; $n_{LP1+FOS}$=103; n_{LP7}=104; $n_{LP7+FOS}$=102; due to missing evaluations of severe lower chest wall indrawing.

Table S3. Adherence to the investigational product (IP) by intervention group, with and without adjustment for enrolment hospital.

	Intervention group						
	**Placebo**	**LP1**	**LP1+FOS**	**LP7**	**LP7+FOS**	***p*[a]**	***p*[b]**
**Participants, N**	104	105	103	104	103		
**Adherence**							
IP doses per infant by 21 days postnatal age, median (25th, 75th)	7 (7,7)	7 (7,7)	7 (7,7)	7 (7,7)	7 (7,7)	0.09	>0.9
IP doses by 21 days postnatal age, n (%)						0.07	0.4
7 doses	99 (95)	102 (97)	103 (100)	103 (99)	102 (99)		
1-6 doses	2 (1.9)	3 (2.9)	0 (0)	1 (1.0)	0 (0)		
Zero doses	3 (2.9)	0 (0)	0 (0)	0 (0)	1 (1.0)		
IP doses by 10 days postnatal age, n (%)						0.2	0.4
7 doses	97 (93)	100 (95)	100 (97)	98 (94)	101 (98)		
1-6 doses	4 (3.8)	5 (4.8)	3 (2.9)	6 (5.8)	1 (1.0)		
Zero doses	3 (2.9)	0 (0)	0 (0)	0 (0)	1 (1.0)		
Timing of first IP dose[c], n (%)						0.8	>0.9
On the day of enrolment	18 (17)	18 (17)	15 (15)	15 (14)	16 (16)		
One day after enrolment	81 (78)	87 (83)	87 (84)	89 (86)	86 (83)		
More than one day after enrolment	2 (1.9)	0 (0)	1 (1.0)	0 (0)	0 (0)		
Age at first IP dose (in days), median (25th, 75th)	2 (1, 3)	2 (1, 3)	2 (1, 3)	2 (1, 3)	2 (1, 2)	>0.9	>0.9
Age at last IP dose (in days), median (25th, 75th)	8 (7, 9)	8 (7, 9)	8 (7, 9)	8 (7, 9)	8 (7, 8)	>0.9	>0.9

[a]p-values were estimated from Kruskal-Wallis tests for ordinal/count variables, and using Fisher's Exact test for categorical variables.

[b]As a post-hoc analysis, p-values were estimated using Wald's test of the coefficients of Poisson regression models, adjusted for hospital enrolment site, for ordinal/count variables, and using Wald's test of coefficients from binomial regression models for categorical variables.

[c]Among infants who received at least one dose of IP.

Table S4. Investigational product (IP) preparation and administration characteristics by intervention group.

	Intervention group				
	**Placebo**	**LP1**	**LP1+FOS**	**LP7**	**LP7+FOS**
Successfully completed IP doses, N	697	725	721	724	714
Solvent used to dilute IP[a], n (%)					
Human milk	536 (77)	581 (80)	550 (76)	576 (80)	558 (78)
Sterile water	53 (7.6)	62 (8.6)	83 (12)	59 (8.1)	76 (11)
Mixture of human milk and sterile water	108 (15)	82 (11)	88 (12)	89 (12)	80 (11)
Location of IP dose administration[a], n (%)					
Hospital	137 (20)	173 (24)	161 (22)	184 (25)	155 (22)
Home	479 (69)	472 (65)	500 (69)	483 (67)	491 (69)
Grandparent's home	79 (11)	76 (10)	55 (7.6)	57 (7.9)	63 (8.8)
Other	2 (0.29)	4 (0.55)	5 (0.69)	0 (0)	5 (0.70)
Location of administration[b] for first IP dose, n (%)					
Hospital	64 (63)	80 (76)	71 (69)	74 (71)	66 (65)
Home	29 (29)	22 (21)	30 (29)	26 (25)	34 (33)
Grandparent's home	8 (7.9)	3 (2.9)	2 (1.9)	4 (3.8)	2 (2.0)
Other	0 (0)	0 (0)	0 (0)	0 (0)	0 (0)
Location of administration[c] for IP doses 2 to 7, n (%)					
Hospital	73 (12)	93 (15)	90 (15)	110 (18)	89 (15)
Home	450 (76)	450 (73)	470 (76)	457 (74)	457 (75)
Grandparent's home	71 (12)	73 (12)	53 (8.6)	53 (8.5)	61 (10)
Other	2 (0.34)	4 (0.65)	5 (0.81)	0 (0)	5 (0.82)
Infants who received at least one IP dose, N	101	105	103	104	102
Proportion of infant's IP doses dissolved in human milk[d], n infants (%)					
100% of IP doses dissolved in human milk	33 (33)	28 (27)	33 (32)	30 (29)	30 (29)
≥50 and <100% of doses dissolved in human milk	58 (57)	61 (59)	61 (59)	67 (64)	62 (61)
<50% doses dissolved in human milk	10 (9.9)	14 (14)	10 (9.6)	8 (7.6)	10 (9.8)

Proportion of infant's IP doses dissolved in water[d], n infants (%)					
100% of doses dissolved in water	0 (0)	0 (0)	1 (0.96)	0 (0)	1 (0.98)
≥50 and <100% of doses dissolved in water	6 (5.9)	4 (3.9)	3 (2.9)	4 (3.8)	2 (2.0)
<50% of doses dissolved in water	95 (94)	99 (96)	100 (96)	101 (96)	99 (97)
Proportion of infant's IP doses dissolved in a mixture of human milk and water[d], n infants (%)					
100% of doses dissolved in a mixture of human milk and water	1 (0.99)	0 (0)	0 (0)	0 (0)	0 (0)
≥50 and <100% of doses dissolved in a mixture of human milk and water	0 (0)	6 (5.8)	2 (1.9)	2 (1.9)	3 (2.9)
<50% of doses dissolved in a mixture of human milk and water	100 (99)	97 (94)	102 (98)	103 (98)	99 (97)
Human milk used as IP solvent, by dose, n infants (%)					
First dose[e]	37 (37)	38 (36)	34 (33)	40 (38)	33 (32)
Second dose[f]	65 (65)	71 (68)	62 (60)	73 (70)	65 (64)
Third dose[g]	74 (74)	86 (83)	79 (77)	83 (80)	87 (85)
Fourth dose[h]	84 (85)	94 (90)	88 (85)	94 (91)	90 (88)
Fifth dose[i]	90 (91)	97 (94)	89 (86)	96 (93)	93 (91)
Sixth dose[j]	92 (93)	98 (96)	101 (98)	92 (89)	94 (92)
Seventh dose[k]	94 (95)	97 (95)	97 (94)	98 (95)	96 (94)

[a]Among successfully completed IP doses

[b]Among successfully completed first IP doses. $N_{Overall}$=515; $N_{Placebo}$=101; N_{LP1}=105; $N_{LP1+FOS}$=103; N_{LP7}=104; $N_{LP7+FOS}$=102

[c]Among successfully completed between 2 to 7 IP doses. $N_{Overall}$=515; $N_{Placebo}$=101; N_{LP1}=105; $N_{LP1+FOS}$=103; N_{LP7}=104; $N_{LP7+FOS}$=102

[d]Among infants who received at least one IP dose. $N_{Overall}$=515; $N_{Placebo}$=101; N_{LP1}=105; $N_{LP1+FOS}$=103; N_{LP7}=104; $N_{LP7+FOS}$=102

[e]Among infants who received their first IP dose. $N_{Overall}$=515; $N_{Placebo}$=101; N_{LP1}=105; $N_{LP1+FOS}$=103; N_{LP7}=104; $N_{LP7+FOS}$=102

[f]Among infants who received their second IP dose. $N_{Overall}$=514; $N_{Placebo}$=100; N_{LP1}=105; $N_{LP1+FOS}$=103; N_{LP7}=104; $N_{LP7+FOS}$=102

[g]Among infants who received their third IP dose. $N_{Overall}$=513; $N_{Placebo}$=100; N_{LP1}=104; $N_{LP1+FOS}$=103; N_{LP7}=104; $N_{LP7+FOS}$=102

[h]Among infants who received their fourth IP dose. $N_{Overall}$=511; $N_{Placebo}$=99; N_{LP1}=104; $N_{LP1+FOS}$=103; N_{LP7}=103; $N_{LP7+FOS}$=102

[i]Among infants who received their fifth IP dose. $N_{Overall}$=510; $N_{Placebo}$=99; N_{LP1}=103; $N_{LP1+FOS}$=103; N_{LP7}=103; $N_{LP7+FOS}$=102

[j]Among infants who received their sixth IP dose. $N_{Overall}$=509; $N_{Placebo}$=99; N_{LP1}=102; $N_{LP1+FOS}$=103; N_{LP7}=103; $N_{LP7+FOS}$=102

[k]Among infants who received their seventh IP dose. $N_{Overall}$=509; $N_{Placebo}$=99; N_{LP1}=102; $N_{LP1+FOS}$=103; N_{LP7}=103; $N_{LP7+FOS}$=102

Table S5. Stool samples collected and analyzed by qPCR by intervention group.

	Intervention group					
	**Placebo**	**LP1**	**LP1+FOS**	**LP7**	**LP7+FOS**	**Total (%)**
All stool samples collected and analyzed by qPCR, n (%)	637 (20)	643 (20)	653 (20)	651 (20)	633 (20)	3217[a] (100)
Post-intervention stool samples collected between days 14 and 60, inclusive, and analyzed by qPCR, n (%)	279 (20)	267 (19)	280 (20)	298 (21)	272 (19)	1396 (100)
Post-intervention stool samples collected between days 14 and 60, inclusive, analyzed by qPCR, and included in primary outcome analyses, n (%)	278 (20)	267 (19)	280 (20)	296 (21)	272 (20)	1393[b] (100)
**Stool samples per infant, n infants (%)[c]**						
1	2 (2.0)	9 (8.7)	6 (5.9)	3 (2.9)	7 (7.0)	27 (5.3)
2	37 (38)	42 (41)	43 (42)	35 (34)	39 (39)	196 (39)
3	38 (39)	35 (34)	30 (29)	39 (38)	33 (33)	175 (35)
4	17 (17)	16 (16)	17 (17)	19 (19)	17 (17)	86 (17)
5	4 (4.1)	1 (0.97)	6 (5.9)	6 (5.9)	4 (4.0)	21 (4.2)
Total number of infants, n	98	103	102	102	100	505 (100)

[a]Six additional stool samples were analyzed by qPCR, despite not being selected randomly or by the algorithm. Five of the samples were from infants in the LP7 group and 1 of the samples was from an infant in the placebo group.

[b]Three infants contributed six samples during the 14 to 60 day window. The prespecified primary outcome was based on a maximum of five post-intervention period stool samples collected per infant during the period and thus, only five samples from each of these infants were randomly selected for inclusion in primary analyses.

[c]Percentage of infants within each intervention group who had a total of 1, 2, 3, 4, or 5 post-intervention stool samples collected between days 14 and 60 and analyzed by qPCR, among all infants with at least one stool sample (N=505).

Table S6. Effect of administration of LP202195 with or without FOS on the absolute abundance of LP202195, relative to placebo or LP7+FOS, using alternative derivations of absolute abundance, alternative timings of sample collection, and per-protocol populations. Absolute abundance in log_{10} cells/μg DNA, except where noted.

	Intervention group				
	**Placebo**	**LP1**	**LP1+FOS**	**LP7**	**LP7+FOS**
**Absolute Abundance (Original)[a]**					
Mean ± SE	1.86 ± 0.03	2.38 ± 0.05	2.58 ± 0.07	3.09 ± 0.07	3.15 ± 0.06
Mean diff (95%CI), vs. placebo	Ref	0.53 (0.41, 0.65)	0.73 (0.59, 0.88)	1.24 (1.09, 1.38)	1.30 (1.16, 1.43)
Mean diff (95%CI), vs. LP7+FOS	-	-0.77 (-0.93, -0.60)	-0.56 (-0.74, -0.38)	-0.06 (-0.24, 0.12)	Ref
**Alternative derivations of Absolute abundance**					
**Alternative Imputation[b]**					
Mean ± SE	1.86 ± 0.03	2.39 ± 0.05	2.58 ± 0.07	3.10 ± 0.06	3.16 ± 0.06
Mean diff (95%CI), vs. placebo	Ref	0.54 (0.42, 0.65)	0.74 (0.59, 0.88)	1.25 (1.11, 1.39)	1.30 (1.17, 1.44)
Mean diff (95%CI), vs. LP7+FOS	-	-0.77 (-0.93, -0.60)	-0.57 (-0.74, -0.39)	-0.05 (-0.23, 0.13)	Ref
**log_{10} cells/g stool**					
Mean ± SE	3.49 ± 0.02	4.05 ± 0.05	4.23 ± 0.06	4.67 ± 0.07	4.72 ± 0.06
Mean diff (95%CI), vs. placebo	Ref	0.57 (0.46, 0.67)	0.75 (0.62, 0.88)	1.19 (1.06, 1.33)	1.23 (1.10, 1.37)
Mean diff (95%CI), vs. LP7+FOS	-	-0.66 (-0.83, -0.50)	-0.48 (-0.66, -0.30)	-0.04 (-0.22, 0.14)	Ref
**log_{10} cells**					
Mean ± SE	0.99 ± 0.02	1.55 ± 0.05	1.73 ± 0.06	2.18 ± 0.07	2.22 ± 0.06
Mean diff (95%CI), vs. placebo	Ref	0.57 (0.46, 0.67)	0.75 (0.61, 0.88)	1.19 (1.05, 1.33)	1.23 (1.10, 1.37)
Mean diff (95%CI), vs. LP7+FOS	-	-0.67 (-0.83, -0.50)	-0.48 (-0.66, -0.30)	-0.04 (-0.22, 0.14)	Ref
**Alternative stool sample collection timings relative to IP administration**					
**Days 42-60: Post-intervention**					
Mean ± SE	1.70 ± 0.04	2.21 ± 0.10	2.44 ± 0.12	2.50 ± 0.11	2.69 ± 0.11
Mean diff (95%CI), vs. placebo	Ref	0.52 (0.32, 0.72)	0.76 (0.51, 1.01)	0.81 (0.59, 1.04)	0.98 (0.76, 1.21)
Mean diff (95%CI), vs. LP7+FOS	-	-0.46 (-0.76, -0.16)	-0.22 (-0.54, 0.09)	-0.17 (-0.47, 0.13)	Ref
**Days 0-60: Pre-, During, and Post-intervention**					
Mean ± SE	1.94 ± 0.03	2.67 ± 0.05	2.79 ± 0.05	3.42 ± 0.06	3.58 ± 0.06

Mean diff (95%CI), vs. placebo	Ref	0.73 (0.63, 0.84)	0.86 (0.75, 0.97)	1.48 (1.35, 1.61)	1.65 (1.51, 1.78)
Mean diff (95%CI), vs. LP7+FOS	-	-0.91 (-1.07, -0.76)	-0.78 (-0.94, -0.63)	-0.16 (-0.34, 0.01)	Ref
**Days 0-60: During and Post-intervention**					
Mean ± SE	1.87 ± 0.03	2.67 ± 0.05	2.83 ± 0.05	3.55 ± 0.06	3.71 ± 0.06
Mean diff (95%CI), vs. placebo	Ref	0.81 (0.70, 0.92)	0.97 (0.86, 1.09)	1.69 (1.56, 1.82)	1.84 (1.71, 1.98)
Mean diff (95%CI), vs. LP7+FOS	-	-1.03 (-1.19, -0.87)	-0.87 (-1.03, -0.71)	-0.16 (-0.34, 0.03)	Ref
**Days 1-22: During intervention**					
Mean ± SE	1.84 ± 0.03	3.24 ± 0.10	3.44 ± 0.10	4.69 ± 0.10	4.75 ± 0.10
Mean diff (95%CI), vs. placebo	Ref	1.40 (1.20, 1.61)	1.59 (1.38, 1.81)	2.84 (2.63, 3.06)	2.91 (2.72, 3.11)
Mean diff (95%CI), vs. LP7+FOS	-	-1.51 (-1.77, -1.24)	-1.32 (-1.60, -1.04)	-0.07 (-0.34, 0.21)	Ref
**Per-protocol analyses**					
**First IP dose within one day of enrolment**					
Mean ± SE	1.83 ± 0.08	2.53 ± 0.12	2.42 ± 0.19	3.19 ± 0.23	2.86 ± 0.17
Mean diff (95%CI), vs. placebo	Ref	0.67 (0.39, 0.95)	0.56 (0.15, 0.98)	1.34 (0.86, 1.81)	1.00 (0.64, 1.36)
Mean diff (95%CI), vs. LP7+FOS	-	-0.33 (-0.74, 0.08)	-0.43 (-0.96, 0.10)	0.34 (-0.19, 0.87)	Ref
**First IP dose by day 2 after birth**					
Mean ± SE	1.90 ± 0.04	2.49 ± 0.06	2.61 ± 0.08	3.01 ± 0.07	3.17 ± 0.07
Mean diff (95%CI), vs. placebo	Ref	0.58 (0.43, 0.72)	0.71 (0.54, 0.88)	1.11 (0.94, 1.27)	1.27 (1.11, 1.43)
Mean diff (95%CI), vs. LP7+FOS	-	-0.69 (-0.88, -0.50)	-0.56 (-0.76, -0.36)	-0.16 (-0.36, 0.04)	Ref
**7 doses of IP by day 10 postnatal age**					
Mean ± SE	1.87 ± 0.03	2.39 ± 0.05	2.60 ± 0.07	3.09 ± 0.07	3.17 ± 0.06
Mean diff (95%CI), vs. placebo	Ref	0.52 (0.40, 0.64)	0.74 (0.60, 0.88)	1.22 (1.08, 1.37)	1.31 (1.17, 1.44)
Mean diff (95%CI), vs. LP7+FOS	-	-0.79 (-0.95, -0.63)	-0.57 (-0.75, -0.38)	-0.08 (-0.27, 0.10)	Ref
**7 doses of IP by day 10 postnatal age & 6 days between first and last IP dose**					
Mean ± SE	1.85 ± 0.03	2.39 ± 0.05	2.60 ± 0.07	3.06 ± 0.07	3.18 ± 0.06
Mean diff (95%CI), vs. placebo	Ref	0.54 (0.42, 0.66)	0.76 (0.62, 0.90)	1.21 (1.06, 1.37)	1.34 (1.20, 1.48)
Mean diff (95%CI), vs. LP7+FOS	-	-0.80 (-0.96, -0.63)	-0.58 (-0.76, -0.39)	-0.13 (-0.30, 0.05)	Ref

[a]These results are identical to the primary analysis presented in Table 2.

[b]Alternative imputation method was used such that the absolute abundance for all samples with a quantification cycle (Cq) value below the lower limit of quantification (LLOQ) of the assay was imputed as half the LLOQ. In the primary analyses shown in Table 2 and under the label ‘Absolute Abundance (Original)’ in this table, cell counts below the LLOQ were imputed based on the values of the standard curve as long as Cq values were below the LLOQ but above the assay’s limit of detection (LOD).

Table S7. Effect of administration of LP202195 with or without FOS on the absolute abundance of LP202195 in stool samples, relative to placebo, by sub-groups. Analyses were conducted using post$_{14-60}$ samples, except where specified.

Intervention group	Mean LP202195 AA, log_{10} cells/μg DNA ± SE		Mean difference in LP202195 AA (95%CI), versus placebo							
	N	Placebo	N	LP1	N	LP1+FOS	N	LP7	N	LP7+FOS
**Primary analysis**	278	1.86 ± 0.03	267	0.53 (0.41, 0.65)	280	0.73 (0.59, 0.88)	296	1.24 (1.09, 1.38)	272	1.30 (1.16, 1.43)
**Mode of delivery**										
Post$_{14-60}$ period										
Vaginal	163	1.89 ± 0.04	133	0.82 (0.65, 1.00)	145	0.91 (0.71, 1.11)	153	0.92 (0.75, 1.10)	148	1.28 (1.10, 1.46)
C-section	115	1.83 ± 0.05	134	0.23 (0.07, 0.39)	135	0.53 (0.34, 0.73)	143	1.56 (1.34, 1.79)	124	1.32 (1.11, 1.54)
During IP period[a]										
Vaginal	87	1.87 ± 0.04	63	1.68 (1.42, 1.95)	74	1.50 (1.25, 1.76)	63	2.88 (2.61, 3.14)	80	2.68 (2.42, 2.95)
C-section	52	1.79 ± 0.05	61	1.12 (0.80, 1.43)	47	1.73 (1.34, 2.12)	52	2.81 (2.50, 3.11)	52	3.33 (3.04, 3.61)
**Study site**										
MFSTC	218	1.92 ± 0.04	196	0.56 (0.42, 0.71)	204	0.71 (0.54, 0.87)	226	1.31 (1.14, 1.48)	212	1.30 (1.14, 1.47)
MCHTI	60	1.66 ± 0.03	71	0.42 (0.24, 0.60)	76	0.79 (0.52, 1.06)	70	1.00 (0.76, 1.25)	60	1.27 (1.02, 1.52)
**Infant feeding pattern**[b]										
Exclusively BF	153	1.89 ± 0.04	176	0.69 (0.54, 0.83)	179	0.71 (0.55, 0.88)	208	1.21 (1.04, 1.38)	195	1.33 (1.17, 1.50)
Not exclusively BF	125	1.82 ± 0.05	91	0.22 (0.03, 0.41)	101	0.75 (0.51, 0.99)	88	1.21 (0.93, 1.48)	77	1.23 (0.97, 1.50)
Predominantly BF	72	1.88 ± 0.05	27	0.48 (0.08, 0.88)	48	0.83 (0.52, 1.14)	44	1.16 (0.79, 1.52)	40	1.10 (0.78, 1.42)
Partially BF	46	1.71 ± 0.09	59	0.19 (-0.07, 0.44)	52	0.74 (0.38, 1.10)	44	1.30 (0.88, 1.73)	33	1.24 (0.78, 1.71)
**Infant antibiotic exposure**[c, d]										
None	219	1.90 ± 0.03	217	0.56 (0.42, 0.70)	249	0.76 (0.61, 0.91)	229	1.41 (1.26, 1.57)	247	1.36 (1.22, 1.51)
Antibiotics	59	1.74 ± 0.05	50	0.32 (0.12, 0.53)	31	0.35 (0.08, 0.62)	67	0.57 (0.36, 0.79)	25	0.72 (0.29, 1.15)

BF, Breastfed.

[a]From day after first IP dose until 24 hours after last IP dose.

[b]Cumulative feeding pattern was derived using data from all clinical visits. For each stool sample, feeding pattern was assigned as the least optimal pattern from the first clinical visit up to the day of stool collection. If there was no clinical visit on the day of the stool collection, the closest visit after the stool collection was selected. If there were no clinical visits after stool collection, the closest preceding clinical visit was used. Predominantly and partially BF groups are a sub-set of the not exclusively BF subgroup. ‘Not BF’ wasn’t included as a sub-group due to small sample size.

[c]Antibiotic exposure was derived using data from all routine clinical visits and visits while infants were ill and/or hospitalized. Antibiotic exposure was defined as ever (vs. never) having antibiotics administered orally or intravenously up until the day of stool collection.

[d]In a post hoc model incorporating infant antibiotic exposure as an interaction with intervention group, and collapsing the two 1-day groups and two 7-day groups to increase sample size across the strata, stool samples collected from infants that were classified as having never having had an antibiotic exposure had an effect estimate that was significantly higher compared to those that did have an exposure in the 7-day combined group (interaction term $p<0.001$), but not in the 1-day combined group ($p=0.07$)).

Table S8. Effect of administration of LP202195 with or without FOS on the absolute abundance of LP202195 in stool samples, relative to LP7+FOS, by sub-groups. Analyses were conducted using $post_{14-60}$ stool samples, except where specified.

	Mean LP202195 AA, log_{10} cells/μg DNA ± SE		Difference in LP202195 AA (95%CI), relative to LP7+FOS					
**Intervention group**	**N**	**LP7+FOS**	**N**	**LP1**	**N**	**LP1+FOS**	**N**	**LP7**
**Primary analysis**	272	3.15 ± 0.06	267	-0.77 (-0.93, -0.60)	280	-0.56 (-0.74, -0.38)	296	-0.06 (-0.24, 0.12)
**Mode of delivery**								
$Post_{14-60}$ period								
Vaginal	148	3.15 ± 0.08	133	-0.46 (-0.69, -0.22)	145	-0.36 (-0.62, -0.11)	153	-0.36 (-0.59, -0.12)
C-section	124	3.18 ± 0.10	134	-1.09 (-1.33, -0.85)	135	-0.79 (-1.05, -0.52)	143	0.24 (-0.03, 0.52)
During IP period[a]								
Vaginal	80	4.55 ± 0.13	63	-1.01 (-1.38, -0.64)	74	-1.18 (-1.53, -0.84)	63	0.19 (-0.17, 0.55)
C-section	52	5.08 ± 0.14	61	-2.22 (-2.64, -1.81)	47	-1.61 (-2.06, -1.17)	52	-0.53 (-0.94, -0.13)
**Study site**								
MFSTC	212	3.22 ± 0.07	196	-0.74 (-0.93, -0.55)	204	-0.59 (-0.80, -0.39)	226	0.01 (-0.20, 0.22)
MCHTI	60	2.93 ± 0.13	71	-0.85 (-1.15, -0.55)	76	-0.48 (-0.83, -0.13)	70	-0.27 (-0.61, 0.08)
**Infant feeding pattern[b]**								
Exclusively BF	195	3.22 ± 0.08	176	-0.65 (-0.84, -0.45)	179	-0.62 (-0.84, -0.40)	208	-0.12 (-0.33, 0.09)
Not exclusively BF	77	3.03 ± 0.13	91	-1.01 (-1.31, -0.72)	101	-0.49 (-0.83, -0.16)	88	-0.04 (-0.39, 0.32)
Predominantly BF	40	2.94 ± 0.16	27	-0.64 (-1.13, -0.15)	48	-0.29 (-0.74, 0.15)	44	0.03 (-0.43, 0.50)
Partially BF	33	2.96 ± 0.22	59	-1.05 (-1.53, -0.57)	52	-0.50 (-1.04, 0.03)	44	0.06 (-0.51, 0.63)
**Infant antibiotic exposure[c]**								
None	247	3.26 ± 0.07	217	-0.80 (-0.98, -0.62)	249	-0.60 (-0.80, -0.41)	229	0.05 (-0.15, 0.25)
Antibiotics	25	2.41 ± 0.20	50	-0.40 (-0.86, 0.05)	31	-0.37 (-0.84, 0.10)	67	-0.15 (-0.62, 0.31)

BF, Breastfed.

[a]Day after first IP dose until 24 hours after last IP dose. Since this analysis was conducted among samples collected during a different time than the primary analyses, it is not a true sub-group analysis.

[b]Cumulative feeding pattern was derived using data from all clinical visits. For each stool sample, feeding pattern was assigned as the least optimal pattern from the first clinical visit up to the day of stool collection. If there was no clinical visit on the day of the stool collection, the closest visit after the stool collection was selected. If there were no clinical visits after stool collection, the closest preceding clinical visit was used. Predominantly and partially BF groups are a sub-set of the not exclusively BF subgroup. 'Not BF' wasn't included as a sub-group due to small sample size.

[c]Antibiotic exposure was derived using data from all routine clinical visits and visits while infants were ill and/or hospitalized. Antibiotic exposure was defined as ever (vs. never) having antibiotics administered orally or intravenously up until the day of stool collection.

Table S9. Absolute abundance of LP202195 at discrete time points following the first dose of investigational product in log cells/μg DNA and log cells/g stool by intervention group

Intervention group	Placebo	LP1	LP1+FOS	LP7	LP7+FOS
Days since first dose	Median AA (log cells/μg DNA) (95%CI)				
8	1.72 (1.59, 1.85)	2.14 (1.85, 2.43)	2.42 (1.99, 2.84)	4.27 (3.95, 4.60)	4.22 (3.62, 4.81)
14	1.85 (1.76, 1.93)	2.10 (1.89, 2.32)	2.24 (1.98, 2.51)	3.14 (2.64, 3.65)	3.37 (2.80, 3.95)
21	1.81 (1.74, 1.88)	2.03 (1.84, 2.22)	2.17 (1.95, 2.40)	2.94 (2.50, 3.37)	3.13 (2.64, 3.62)
28	1.77 (1.71, 1.84)	1.96 (1.79, 2.13)	2.10 (1.91, 2.28)	2.73 (2.37, 3.09)	2.89 (2.48, 3.30)
60	1.60 (1.49, 1.71)	1.63 (1.43, 1.82)	1.75 (1.63, 1.88)	1.78 (1.62, 1.93)	1.78 (1.61, 1.95)
90	1.55 (1.45, 1.64)	1.59 (1.44, 1.74)	1.71 (1.62, 1.81)	1.73 (1.62, 1.85)	1.73 (1.59, 1.86)
180	1.39 (1.17, 1.62)	1.48 (1.39, 1.57)	1.59 (1.45, 1.74)	1.59 (1.49, 1.70)	1.57 (1.48, 1.67)
Days since first dose	Median AA (log cells/g stool) (95%CI)				
8	3.39 (3.37, 3.41)	3.46 (3.17, 3.76)	3.79 (3.05, 4.53)	5.99 (5.52, 6.47)	5.83 (5.17, 6.49)
14	3.39 (3.37, 3.41)	3.45 (3.41, 3.49)	3.44 (3.32, 3.57)	4.50 (3.79, 5.20)	5.00 (4.25, 5.75)
21	3.39 (3.37, 3.40)	3.44 (3.41, 3.47)	3.44 (3.33, 3.55)	4.33 (3.73, 4.92)	4.76 (4.12, 5.39)
28	3.38 (3.37, 3.40)	3.43 (3.41, 3.46)	3.43 (3.35, 3.52)	4.16 (3.67, 4.65)	4.51 (3.99, 5.04)
60	3.38 (3.36, 3.40)	3.39 (3.37, 3.42)	3.41 (3.38, 3.44)	3.38 (3.35, 3.41)	3.41 (3.37, 3.44)
90	3.39 (3.37, 3.41)	3.39 (3.37, 3.41)	3.41 (3.39, 3.43)	3.39 (3.36, 3.41)	3.40 (3.38, 3.42)
180	3.42 (3.39, 3.46)	3.39 (3.37, 3.42)	3.41 (3.39, 3.43)	3.40 (3.38, 3.41)	3.38 (3.35, 3.42)

Table S10. Routine biochemistry test results by intervention group.

Analyte	Age (days)[a]	Summary statistic[b,c]	Intervention group: Placebo	LP1	LP1+FOS	LP7	LP7+FOS	p^{d}
Number of infants[e]	9	N	35	44	39	44	38	-
	60	N	56	46	53	47	50	-
Procalcitonin (ng/mL)	9	Median (25th, 75th)	0.07 (0.06, 0.09)	0.07 (0.06, 0.09)	0.08 (0.06, 0.08)	0.06 (0.05, 0.08)	0.06 (0.05, 0.08)	0.2
		OOR, n (%)	0 (0)	0 (0)	0 (0)	0 (0)	0 (0)	-
	60	Median (25th, 75th)	0.06 (0.04, 0.07)	0.06 (0.04, 0.09)	0.06 (0.04, 0.07)	0.06 (0.05, 0.07)	0.06 (0.05, 0.07)	0.9
		OOR, n (%)	0 (0)	0 (0)	1 (1.9)	0 (0)	0 (0)	>0.9
High-sensitivity CRP (mg/L)	9	Median (25th, 75th)	0.17 (0.11, 0.29)	0.32 (0.18, 0.57)	0.23 (0.18, 0.58)	0.21 (0.13, 0.48)	0.23 (0.13, 0.38)	0.1
		OOR, n (%)	1 (2.9)	0 (0)	1 (2.6)	0 (0)	0 (0)	0.3
	60	Median (25th, 75th)	0.45 (0.22, 0.92)	0.48 (0.21, 0.79)	0.50 (0.26, 0.92)	0.46 (0.25, 1.00)	0.41 (0.29, 0.93)	>0.9
		OOR, n (%)	14 (25)	11 (24)	14 (26)	13 (28)	13 (26)	0.3
Creatinine (µmol/L)	9	Mean (SD)	34 (6.6)	36 (6.3)	34 (6.3)	36 (8.0)	36 (10)	0.6
		OOR, n (%)	2 (5.7)	1 (2.3)	1 (2.6)	1 (2.3)	1 (2.6)	0.9
	60	Mean (SD)	22 (4.8)	21 (4.8)	21 (4.1)	21 (3.4)	21 (3.8)	0.9
		OOR, n (%)	5 (8.9)	3 (6.5)	3 (5.7)	2 (4.3)	2 (4.0)	0.9
Alanine transaminase (U/L)	9	Median (25th, 75th)	17 (13, 25)	19 (13, 24)	17 (13, 21)	17 (11, 23)	19 (12, 23)	>0.9
		OOR, n (%)	3 (8.6)	4 (9.1)	4 (10)	7 (16)	4 (11)	0.9
	60	Median (25th, 75th)	32 (25, 44)	29 (22, 45)	29 (20, 48)	30 (24, 46)	34 (24, 47)	0.7
		OOR, n (%)	31 (55)	22 (48)	25 (47)	24 (51)	30 (60)	0.7
Total bilirubin (µmol/L)	9	Median (25th, 75th)	133 (84, 171)	141 (98, 198)	132 (83, 201)	124 (77, 182)	133 (91, 175)	0.9
		OOR, n (%)	3 (8.6)	2 (4.5)	3 (7.7)	5 (11)	1 (2.6)	0.6
	60	Median (25th, 75th)	12 (8, 15)	12 (8, 19)	10 (7, 16)	12 (9, 20)	13 (9, 18)	0.5

		OOR, n (%)	30 (54)	23 (50)	21 (40)	24 (51)	26 (52)	0.6
Direct bilirubin (μmol/L)	9	Median (25th, 75th)	11 (9, 13)	11 (9, 13)	10 (8, 13)	11 (9, 13)	10 (8, 13)	0.8
		OOR, n (%)	29 (83)	36 (82)	29 (74)	37 (84)	33 (87)	0.7
	60	Median (25th, 75th)	2.3 (1.6, 3.4)	2.3 (1.7, 4.6)	2.1 (1.6, 3.3)	2.6 (1.9, 4.0)	2.9 (1.8, 3.9)	0.2
		OOR, n (%)	20 (36)	15 (33)	16 (30)	19 (40)	24 (48)	0.7
Indirect bilirubin (μmol/L)	9	Median (25th, 75th)	121 (73, 157)	132 (89, 185)	123 (75, 186)	114 (67, 172)	124 (81, 159)	0.9
		OOR, n (%)	22 (63)	29 (66)	25 (64)	24 (55)	26 (68)	0.7
	60	Median (25th, 75th)	9.5 (6.8, 12.1)	8.7 (5.8, 14.6)	7.9 (5.6, 13.2)	9.2 (6.7, 15.1)	10 (7, 15)	0.6
		OOR, n (%)	19 (35)	20 (43)	15 (28)	19 (40)	22 (44)	0.7
Albumin (g/L)	9	Mean (SD)	35 (2.2)	36 (3.0)	36 (2.8)	36 (2.2)	36 (2.7)	0.8
		OOR, n (%)	0 (0)	2 (4.5)	1 (2.6)	0 (0)	1 (2.6)	0.7
	60	Mean (SD)	40 (2.1)	39 (2.9)	39 (3.2)	39 (2.4)	39 (3.8)	0.5
		OOR, n (%)	0 (0)	0 (0)	1 (1.9)	0 (0)	2 (4.0)	0.7
Glucose (mmol/L)	9	Mean (SD)	4.8 (0.74)	4.6 (0.58)	4.4 (0.47)	4.7 (0.77)	4.7 (0.60)	0.09
		OOR, n (%)	7 (21)	3 (7.0)	0 (0)	5 (12)	5 (14)	0.05[f]
	60	Mean (SD)	5.2 (0.39)	5.2 (0.47)	5.1 (0.45)	5.1 (0.45)	5.1 (0.58)	0.6
		OOR, n (%)	1 (1.8)	2 (4.3)	1 (1.9)	1 (2.1)	3 (6.0)	0.05[f]

OOR, Out of range.

[a]Median (25th, 75th) age at sample collection for routine day 9 and 60 samples was 9 (9, 11) and 61 (60, 66), respectively.

[b]Age specific reference ranges were adapted from the SickKids lab services guide and the CALIPER cohort database (DOI: 10.5683/SP3/3QNINX).

[c]For procalcitonin, high-sensitivity CRP, creatinine, alanine transaminase, and bilirubin concentrations, only values that fell above the upper limit of the reference range were reported as outside of the range.

[d]p-values for differences in analyte concentrations across groups (adjusted for site) were generated using linear regression models (see text for details). The concentrations of alanine transaminase, direct bilirubin, high-sensitivity CRP, indirect bilirubin, procalcitonin, and total bilirubin were log-transformed prior to generated p-values. P-values for differences in the proportion of infants with analyte concentrations outside of the reference range across groups were generated using permutation testing (see text for details).

[e]See S14 for sample sizes contributing to each analyte and timepoint, by intervention group.

[f]After correction for multiple testing using the Holm procedure, the p-value was not statistically significant.

Table S11. Routine hematology test results by intervention group.

Analyte	Age (days)[a]	Summary statistic[b,c]	Intervention group					p[d]
			Placebo	LP1	LP1+FOS	LP7	LP7+FOS	
Number of infants[e]	9	N	34	43	39	44	38	-
	60	N	56	46	52	47	49	-
Hemoglobin (g/dL)	9	Mean (SD)	16 (1.6)	16 (1.8)	16 (1.7)	16 (1.9)	16 (1.6)	0.4
		OOR, n (%)	3 (8.8)	2 (4.6)	1 (2.6)	5 (11)	3 (7.9)	0.6
	60	Mean (SD)	11 (1.0)	10 (1.2)	10 (0.88)	10 (0.81)	10 (1.1)	0.5
		OOR, n (%)	3 (5.4)	9 (20)	5 (9.6)	6 (13)	7 (14)	0.3
Hematocrit (%)	9	Mean (SD)	46 (4.7)	48 (5.3)	47 (5.1)	46 (5.7)	47 (4.6)	0.3
		OOR, n (%)	3 (8.8)	2 (4.6)	1 (2.6)	6 (14)	1 (2.6)	0.2
	60	Mean (SD)	31 (3.1)	31 (3.7)	31 (2.7)	31 (2.6)	31 (3.5)	0.8
		OOR, n (%)	7 (12)	11 (24)	7 (13)	11 (23)	10 (20)	0.4
Red blood cells (10^{12}/L)	9	Mean (SD)	4.6 (0.45)	4.9 (0.57)	4.8 (0.51)	4.8 (0.58)	4.8 (0.47)	0.4
		OOR, n (%)	6 (18)	4 (9.3)	1 (2.6)	4 (9.1)	2 (5.3)	0.2
	60	Mean (SD)	3.6 (0.43)	3.6 (0.58)	3.6 (0.36)	3.5 (0.35)	3.6 (0.41)	0.9
		OOR, n (%)	1 (1.8)	3 (6.5)	2 (3.8)	1 (2.1)	1 (2.0)	0.7
Mean corpuscular volume (fl)	9	Mean (SD)	99 (4.2)	99 (5.5)	99 (3.9)	98 (5.8)	99 (4.4)	0.6
		OOR, n (%)	5 (15)	11 (26)	3 (7.7)	13 (30)	6 (16)	0.1
	60	Mean (SD)	87 (4.5)	86 (6.2)	87 (5.8)	87 (5.4)	86 (5.6)	0.4
		OOR, n (%)	2 (3.6)	6 (13)	6 (12)	5 (11)	3 (6.1)	0.4
Mean corpuscular hemoglobin (pg)	9	Mean (SD)	34 (1.4)	34 (1.9)	34 (1.3)	33 (1.9)	34 (1.6)	0.3
		OOR, n (%)	5 (15)	11 (26)	3 (7.7)	12 (27)	8 (21)	0.2
	60	Mean (SD)	30 (1.7)	29 (2.3)	29 (2.0)	29 (2.0)	29 (2.3)	0.2
		OOR, n (%)	1 (1.8)	5 (11)	4 (7.7)	4 (8.5)	5 (10)	0.5
Total leukocyte count (10^9/L)	9	Mean (SD)	11 (2.6)	12 (2.4)	12 (2.4)	12 (2.7)	11 (2.2)	0.3
		OOR, n (%)	4 (12)	8 (19)	6 (15)	8 (18)	5 (13)	>0.9
	60	Mean (SD)	11 (2.4)	11 (2.8)	12 (3.2)	11 (3.3)	12 (2.8)	0.8

		OOR, n (%)	9 (16)	8 (17)	15 (29)	12 (26)	9 (18)	0.5
Neutrophils (10^9/L)	9	Mean (SD)	3.9 (1.9)	4.2 (2.1)	4.0 (1.2)	4.3 (1.5)	3.9 (1.3)	0.7
		OOR, n (%)	4 (12)	3 (7.0)	1 (2.6)	5 (11)	1 (2.6)	0.3
	60	Mean (SD)	2.4 (0.99)	2.2 (1.0)	2.4 (1.0)	2.4 (1.4)	2.6 (1.2)	0.7
		OOR, n (%)	2 (3.6)	3 (6.5)	2 (3.8)	1 (2.1)	1 (2.0)	0.8
Lymphocytes (10^9/L)	9	Mean (SD)	5.9 (1.1)	5.8 (1.5)	6.4 (1.6)	6.3 (1.6)	5.6 (1.4)	0.04[f]
		OOR, n (%)	2 (5.9)	4 (9.3)	8 (21)	8 (18)	1 (2.6)	0.06
	60	Mean (SD)	8.0 (1.8)	8.3 (2.3)	8.5 (2.4)	8.1 (2.6)	8.0 (2.4)	0.8
		OOR, n (%)	20 (36)	19 (41)	20 (38)	13 (28)	16 (33)	0.7
Monocytes (10^9/L)	9	Mean (SD)	1.1 (0.35)	1.0 (0.25)	0.98 (0.27)	1.0 (0.28)	1.0 (0.37)	0.8
		OOR, n (%)	1 (2.9)	1 (2.3)	0 (0)	2 (4.5)	2 (5.3)	0.7
	60	Mean (SD)	0.62 (0.22)	0.57 (0.22)	0.60 (0.21)	0.56 (0.22)	0.57 (0.19)	0.7
		OOR, n (%)	2 (3.6)	2 (4.3)	3 (5.8)	4 (8.5)	1 (2.0)	0.7
Eosinophils (10^9/L)	9	Mean (SD)	0.47 (0.26)	0.53 (0.34)	0.45 (0.21)	0.50 (0.26)	0.50 (0.29)	0.8
		OOR, n (%)	6 (18)	12 (28)	4 (10)	8 (18)	6 (16)	0.3
	60	Mean (SD)	0.36 (0.17)	0.30 (0.19)	0.43 (0.31)	0.32 (0.17)	0.38 (0.22)	0.03[f]
		OOR, n (%)	4 (7.1)	1 (2.2)	7 (13)	0 (0)	3 (6.1)	0.06
Basophils (10^9/L)	9	Mean (SD)	0.05 (0.03)	0.06 (0.03)	0.06 (0.02)	0.06 (0.02)	0.07 (0.04)	0.2
		OOR, n (%)	6 (18)	4 (9.3)	2 (5.1)	8 (18)	7 (18)	0.3
	60	Mean (SD)	0.03 (0.02)	0.04 (0.02)	0.04 (0.02)	0.04 (0.02)	0.04 (0.02)	0.7
		OOR, n (%)	7 (12)	6 (13)	11 (21)	9 (19)	5 (10)	0.5
Platelet count (10^9/L)	9	Mean (SD)	364 (104)	392 (103)	398 (140)	393 (102)	371 (111)	0.6
		OOR, n (%)	9 (26)	14 (33)	16 (41)	19 (43)	11 (29)	0.5
	60	Mean (SD)	465 (133)	455 (157)	459 (148)	464 (126)	489 (123)	0.7
		OOR, n (%)	9 (16)	9 (20)	11 (21)	8 (17)	9 (18)	>0.9

OOR, out of range.

[a] Median (25th, 75th) age at sample collection for routine day 9 and 60 samples was 9 (9, 11) and 61 (60, 66), respectively.

[b]Age specific reference ranges were adapted from the SickKids lab services guide and the CALIPER cohort database (DOI: 10.5683/SP3/3QNINX).

[c]For hemoglobin, hematocrit, and red blood cell concentrations, only values that fell below the lower limit of the reference range were reported as outside of the range.

[d]p-values for differences in analyte concentrations across groups (adjusted for site) were generated using linear regression models (see text for details). p-values for differences in the proportion of infants with analyte concentrations outside of the reference range across groups were generated using permutation testing (see text for details).

[e]See Table S14 for sample sizes contributing to each analyte and timepoint, by intervention group.

[f]After correction for multiple testing using the Holm procedure, the p-value was not statistically significant.

Table S12. Adverse events in the post-investigational product (IP) administration period by intervention group.

	Intervention group					
	**Placebo**	**LP1**	**LP1+FOS**	**LP7**	**LP7+FOS**	***p*[b]**
**Participants, N[a]**	**100**	**104**	**103**	**103**	**102**	
**Caregiver-reported symptoms in the post-IP administration period up to 60 days, inclusive, n[c]**						
Time at risk (days)[d]	4902	5039	5047	4999	5007	
Abdominal distension	26	12	39	27	7	0.7
Abdominal gas/flatulence	5	0	5	4	5	0.4
Acute diarrhea	2	16	8	18	14	0.4
Persistent vomiting (≥ 3 times in 24 hours)[e]	4	6	5	9	0	0.2
Projectile vomiting[e]	0	6	13	9	2	0.6
Vomiting	24	37	26	28	5	0.5
Not gaining enough weight[f]	0	1	0	2	2	0.7
Poor feeding	0	5	2	0	0	>0.9
Red or discharging umbilicus	11	9	47	23	4	0.2
Skin pustules or boil	22	11	0	23	20	0.5
Unusual skin rash or anything abnormal on skin	30	34	8	52	16	0.1
Yellowing of skin or eyes	7	12	1	10	0	0.4
Drainage from ear	7	0	0	0	4	0.3
Red/oozing/swollen eyes	17	38	61	36	20	0.5
Sores inside mouth	1	18	14	6	11	0.8
White or grey patches or coating inside mouth	0	0	2	0	0	0.8
Cough	85	147	121	100	76	0.4
Fast or difficult breathing	6	0	4	0	7	0.4
Runny nose	50	17	16	17	6	0.05[g]
Stuffy nose	132	168	215	209	123	0.3
Cold to the touch or has low body temperature	0	1	0	0	1	0.8
Hot to the touch or has fever	25	29	21	10	27	0.4
Unusually sleepy or could not wake from sleep	0	5	0	0	0	>0.9

Other symptoms[h]	7	2	2	3	1	0.3
**Caregiver-reported symptoms in the post-IP administration period from >60 days to 180 days, n[c]**						
Time at risk (days)[i]	13098	12928	12961	12718	12894	
Abdominal distension	2	0	2	0	0	0.4
Acute diarrhea	23	29	8	31	12	0.4
Persistent vomiting (≥ 3 times in 24 hours)[e]	1	2	1	1	1	>0.9
Projectile vomiting[e]	0	0	2	0	0	0.8
Vomiting	4	7	2	3	1	0.7
Poor feeding	0	6	3	0	0	>0.9
Skin pustules or boil	4	0	12	8	7	>0.9
Unusual skin rash or anything abnormal on skin	21	17	0	5	10	0.5
Drainage from ear	0	8	0	0	0	>0.9
Red/oozing/swollen eyes	5	23	2	0	10	0.3
Sores inside mouth	0	24	0	6	5	0.1
Cough	144	146	94	80	57	0.1
Fast or difficult breathing	0	3	8	5	0	0.8
Runny nose	59	77	55	51	49	0.8
Stuffy nose	88	81	88	47	63	0.6
Cold to the touch or has low body temperature	0	2	0	0	3	0.6
Hot to the touch or has fever	38	43	24	27	14	0.6
Other symptoms[h]	1	2	0	0	3	0.5
**Community health research worker-observed signs in the post-IP administration period up to 60 days, n (%)[j]**						
Number of visits at which an infant was examined[k]	714	721	746	744	718	
Poor feeding (not sucking effectively)[l,m]	0 (0)	3 (0.4)	0 (0)	1 (0.1)	0 (0)	0.2
Jaundice[n]	1 (0.1)	3 (0.4)	1 (0.1)	3 (0.4)	1 (0.1)	0.8
Skin pustules or abscess	2 (0.3)	5 (0.7)	1 (0.1)	4 (0.5)	3 (0.4)	0.6
Skin rash	4 (0.6)	7 (1)	0 (0)	3 (0.4)	0 (0)	0.02[g]

Umbilicus red, discoloured or discharging pus[o]	1 (0.1)	1 (0.1)	4 (0.5)	4 (0.5)	0 (0)	0.5
Discharge from ear	1 (0.1)	0 (0)	0 (0)	0 (0)	1 (0.1)	0.3
Sunken, red, oozing, or swollen eyes[p]	2 (0.3)	0 (0)	2 (0.3)	2 (0.3)	0 (0)	0.6
Oral thrush (clinical diagnosis)	0 (0)	0 (0)	2 (0.3)	0 (0)	0 (0)	0.2
Ulcers in mouth	0 (0)	0 (0)	1 (0.1)	1 (0.1)	1 (0.1)	>0.9
Audible wheeze or whistling breath sounds	3 (0.4)	0 (0)	0 (0)	1 (0.1)	0 (0)	0.03[g]
Cough	7 (1.0)	9 (1.2)	6 (0.8)	9 (1.2)	1 (0.1)	0.2
Nasal discharge/rhinorrhea	2 (0.3)	1 (0.1)	0 (0)	1 (0.1)	3 (0.4)	0.3
Severe lower chest wall indrawing[l,q]	0 (0)	2 (0.3)	0 (0)	1 (0.1)	0 (0)	0.5
Weak, abnormal, or absent cry	1 (0.1)	3 (0.4)	0 (0)	0 (0)	0 (0)	0.04[g]
Fever (≥37.5°C)[l]	1 (0.1)	0 (0)	0 (0)	0 (0)	0 (0)	0.2
Hypothermia (<35.5°C)[l]	1 (0.1)	0 (0)	0 (0)	1 (0.1)	0 (0)	0.6
Convulsions[l,n]	0 (0)	0 (0)	0 (0)	0 (0)	0 (0)	-
No movement, movement only with stimulation, or unconscious[l]	0 (0)	1 (0.1)	0 (0)	0 (0)	0 (0)	0.4
Other signs of illness[r]	2 (0.3)	0 (0)	3 (0.4)	4 (0.5)	1 (0.1)	0.4
**Community health research worker-observed signs in the post-IP administration period from >60 days to 180 days, n (%)[j]**						
Number of visits at which an infant was examined[s]	218	238	229	230	219	
Poor feeding (not sucking effectively)[l,t]	0 (0)	0 (0)	0 (0)	0 (0)	0 (0)	-
Jaundice	0 (0)	0 (0)	2 (0.9)	0 (0)	0 (0)	0.1
Skin pustules or abscess	0 (0)	0 (0)	1 (0.4)	1 (0.4)	0 (0)	0.7
Skin rash	1 (0.5)	0 (0)	0 (0)	2 (0.9)	2 (0.9)	0.4
Sunken, red, oozing, or swollen eyes[u]	0 (0)	1 (0.4)	0 (0)	0 (0)	1 (0.5)	0.7
Ulcers in mouth	0 (0)	2 (0.8)	0 (0)	1 (0.4)	0 (0)	0.5
Audible wheeze or whistling breath sounds	1 (0.5)	0 (0)	1 (0.4)	0 (0)	1 (0.5)	0.6
Cough	7 (3.2)	5 (2.1)	2 (0.9)	6 (2.6)	4 (1.8)	0.5
Elevated respiratory rate (≥50 breaths/min)	4 (1.8)	4 (1.7)	10 (4.4)	8 (3.5)	2 (0.9)	0.1
Nasal discharge/rhinorrhea	2 (0.9)	4 (1.7)	1 (0.4)	2 (0.9)	5 (2.3)	0.4

Severe lower chest wall indrawing[l]	0 (0)	0 (0)	0 (0)	0 (0)	1 (0.5)	0.2
Fever (≥37.5°C)[l]	1 (0.5)	0 (0)	1 (0.4)	1 (0.4)	1 (0.5)	0.8
Hypothermia (<35.5°C)[l]	0 (0)	0 (0)	0 (0)	0 (0)	0 (0)	-
Convulsions[l,v]	0 (0)	0 (0)	0 (0)	0 (0)	0 (0)	-
No movement, movement only with stimulation, or unconscious[l]	0 (0)	0 (0)	0 (0)	0 (0)	0 (0)	-
Other signs of illness[r]	2 (0.9)	0 (0)	0 (0)	0 (0)	0 (0)	0.02[g]

[a]Infants who received at least one dose of the investigational product (IP) and contributed at least one day of time at risk in the post IP period.

[b]p-values were based on permutation testing (see text for details).

[c]The number of calendar days on which a caregiver reported an infant had symptoms.

[d]Defined as the number of calendar days starting from 3 days after the final IP dose up to 60 days of age, or until the infant exited the study, whichever came first.

[e]Persistent and projectile vomiting events are a sub-set of vomiting events.

[f]Count of the number of visits at which the caregiver indicated the symptom was present.

[g]No longer significant after the Holm's procedure.

[h]Other symptoms in the post-IP period up to 60 days included blood in stool, ≥6 hours since last passed urine, abnormal movement (convulsions/fits), abdominal gas/flatulence, noisy breathing, chest congestion, constipation, and other symptoms that we were unable to classify. In the post-IP period > 60 days to 180 days other symptoms included constipation, dandruff, swelling, abdominal gas/flatulence, not gaining enough weight, and abnormal movement (convulsions/fits).

[i]Defined as the number of calendar days starting from 61 days of age up to 6 months of age, or until the infant exited the study. The overall median age (25th, 75th) at study exit was 181 days (180 days, 187 days).

[j]Count (n) and percentage (%) of visits at which the sign was observed among all visits at which the relevant examination was conducted.

[k]Among the 508 infants that were examined at least once by a community health research worker during this period.

[l]Sign of clinical severe infection.

[m]$n_{Overall}$=3619; $n_{Placebo}$=741; n_{LP1}=714; $n_{LP1+FOS}$=709; n_{LP7}=716; $n_{LP7+FOS}$=739; due to missing evaluations of poor feeding (not suckling effectively).

[n]$n_{Overall}$=3622; $n_{Placebo}$=741; n_{LP1}=715; $n_{LP1+FOS}$=710; n_{LP7}=717; $n_{LP7+FOS}$=739; due to missing evaluations of jaundice/convulsions.

[o] $n_{Overall}$=3622; $n_{Placebo}$=740; n_{LP1}=715; $n_{LP1+FOS}$=710; n_{LP7}=718; $n_{LP7+FOS}$=739; due to missing evaluations of umbilicus red, discoloured or discharging pus.

[p] $n_{Overall}$=3617; $n_{Placebo}$=739; n_{LP1}=714; $n_{LP1+FOS}$=709; n_{LP7}=718; $n_{LP7+FOS}$=737; due to missing evaluations of sunken, red, oozing, or swollen eyes.

[q] $n_{Overall}$=3621; $n_{Placebo}$=741; n_{LP1}=714; $n_{LP1+FOS}$=710; n_{LP7}=717; $n_{LP7+FOS}$=739; due to missing evaluations of severe lower chest wall indrawing.

[r] Other signs in the post-IP period up to 60 days included skin pinch, elevated respiratory rate, abdominal gas, constipation, vomiting, swelling, inflamed belly button (no pus or redness), and nose sounds. In the post-IP period up >60 days to 180 days other signs included drainage in ear and hemangioma.

[s] Among 491 infants who were examined at least once by a community health research worker during this period.

[t] $n_{Overall}$=1114; $n_{Placebo}$=227; n_{LP1}=215; $n_{LP1+FOS}$=213; n_{LP7}=232; $n_{LP7+FOS}$=227; due to missing evaluations of poor feeding (not suckling effectively).

[u] $n_{Overall}$=1112; $n_{Placebo}$=227; n_{LP1}=216; $n_{LP1+FOS}$=213; n_{LP7}=230; $n_{LP7+FOS}$=226; due to missing evaluations of sunken, red, oozing, or swollen eyes.

[v] $n_{Overall}$=1115; $n_{Placebo}$=227; n_{LP1}=216; $n_{LP1+FOS}$=214; n_{LP7}=232; $n_{LP7+FOS}$=226; due to missing evaluations of convulsions.

Table S13. Adverse events reported or observed during ad hoc medical assessments of non-hospitalized infants in the period beyond the baseline assessment and up to 6 months by intervention group.

	Intervention group					
	**Placebo**	**LP1**	**LP1+FOS**	**LP7**	**LP7+FOS**	**P[b]**
**Participants, N[a]**	**105**	**103**	**104**	**102**	**101**	
Ad hoc outpatient events[c], n	502	384	413	441	324	0.2
In-person[d], n	107	94	84	98	67	0.2
Over the phone[e], n	402	291	329	346	258	0.2
Infants with at least one ad hoc outpatient event[f], n (%)	61 (60)	66 (63)	57 (55)	66 (63)	58 (57)	0.7
Infants with at least one in-person ad hoc outpatient event[d], n (%)	48 (48)	48 (46)	45 (44)	52 (50)	45 (44)	0.9
Infants with at least one over the phone outpatient event[e], n (%)	60 (59)	61 (58)	57 (55)	64 (62)	56 (55)	0.9
**Caregiver-reported symptoms up to 180 days, n (%)[g]**						
Time at risk (days)[h]	18004	18423	18536	18280	18356	
Abdominal distension	29	20	23	54	40	0.6
Acute diarrhea	79	55	104	63	78	0.5
Blood in stool	0	0	4	0	0	0.1
Projectile vomiting[i]	0	5	3	0	0	>0.9
Vomiting	34	41	19	22	3	0.4
Not gaining enough weight	0	8	1	0	0	>0.9
Poor feeding	1	9	0	0	3	0.8
≥ 6 hours since last passed urine	0	0	1	1	0	0.8
Red or discharging umbilicus	31	14	51	41	8	0.2
Skin pustules or boil	9	3	12	37	8	0.3
Unusual skin rash or anything abnormal on skin	124	80	40	112	33	0.2
Yellowing of skin or eyes	19	75	38	64	32	0.3
Drainage from ear	9	3	0	6	3	0.8
Red/oozing/swollen eyes	55	57	39	65	42	>0.9
Sores inside mouth	5	33	4	6	0	0.5
White or grey patches or coating inside mouth	1	26	20	0	0	0.05[j]

Cough	383	365	301	315	234	0.5
Fast or difficult breathing	6	13	0	0	1	0.2
Runny nose	135	64	32	78	60	0.02[j]
Stuffy nose	202	231	212	181	166	0.8
Cold to the touch or has low body temperature	0	0	0	0	0	-
Hot to the touch or has fever	60	55	23	32	40	0.1
Abnormal movement (convulsions/fits)	0	1	0	0	0	>0.9
Weak, abnormal, or absent cry	5	11	1	1	0	0.7
Other signs of illness[k]	27	19	39	23	16	0.4
**Study personnel-observed signs up to 180 days, n (%)[l]**						
Poor feeding (not sucking effectively)[m]	0	0	0	0	0	-
Jaundice	5	10	8	13	5	0.4
Skin pustules or abscess	2	0	1	1	1	0.7
Skin rash	9	4	6	8	3	0.4
Umbilicus red, discoloured or discharging pus	5	2	3	5	1	0.4
Discharge from ear	2	0	0	0	0	0.04[j]
Sunken, red, oozing, or swollen eyes	4	7	9	2	2	0.1
Oral thrush	2	7	1	0	0	0.03[j]
Audible wheeze or whistling breath sounds	1	0	1	0	0	0.4
Cough	40	37	26	42	25	0.3
Elevated respiratory rate (≥60 breaths/min)[n]	0	0	0	0	1	0.4
Nasal discharge/rhinorrhea	29	35	24	29	20	0.5
Severe lower chest wall indrawing[m]	0	0	0	0	1	0.4
Weak, abnormal, or absent cry	0	0	0	1	0	0.8
Fever (≥37.5°C)[m]	8	4	1	1	4	0.05[j]
Hypothermia (<35.5°C)[m]	0	0	0	1	0	0.8
Convulsions[m]	0	0	0	0	0	-
No movement, movement only with stimulation, or unconscious[m]	0	0	0	0	0	-

Other signs of illness[o]	3	3	3	4	4	>0.9

[a]Infants that received at least one dose of the investigational product and contributed at least one day "at-risk" in the period beyond the baseline assessment and up to 6 months or study exit, whichever came first.

[b]p-values for across-group differences were based on permutation testing (see text for details).

[c]Among all ad hoc medical assessments, if more than one ad hoc medical assessment occurred per participant per calendar day, assessments were collapsed into a single event per infant per day, inclusive of all signs/symptoms that were reported at any one of the visits; baseline assessments are not included but are reported in Table S2.

[d]Among all in-person ad hoc assessments, if more than one in-person ad hoc medical assessment occurred per participant per calendar day, in-person assessments were collapsed into a single event per infant per day, inclusive of all signs/symptoms that were reported at any one of the visits.

[e]Among all over the phone ad hoc medical assessments, if more than one over the phone ad hoc medical assessment occurred per participant per calendar day, over the phone assessments were collapsed into a single event per infant per day, inclusive of all signs/symptoms that were reported at any one of the visits.

[f]Among all infants with at least one ad hoc medical assessment, if more than one ad hoc medical assessment occurred per participant per calendar day, assessments were collapsed into a single event per infant per day, inclusive of all signs/symptoms that were reported at any one of the visits; baseline assessments are not included but are reported in Table S2.

[g]The number of calendar days on which a caregiver reported an infant had symptoms.

[h]The number of calendar days starting from the day after the baseline assessment up to 6 months, or until the infant exited the study, whichever came first.

[i]Projectile vomiting events are a sub-set of vomiting events.

[j]P-values no longer significant after the Holm's procedure.

[k]Other signs of illness observed by the caregiver include abdominal cramping or pain, abdominal gas, flatulence, regurgitation, constipation, mucoid stool, frothy stool, yellowish semisolid stool, greenish stool, hard stool, pinworms, discomfort while urinating, double urethral opening, noisy breathing, discharge from wound, lesion in scalp, lethargy, feeding issues, bluish coloration when crying, swelling of body parts, itching in ear, infantile colic, nasal bleeding, vaginal bleeding, and other umbilical concern.

[l]The number of calendar days on which a study medical officer observed an infant had signs.

[m]Sign of clinical severe infection.

[n]For infants older than 60 days postnatal age, the threshold for elevated respiratory rate was ≥ 50 breaths per minute; however, there were no instances where infants met this criterion.

[o]Other signs of illness observed by the study medical officer included abdominal distension, feeding issue, soft tissue swelling, noisy breathing, cardiac concern, blood in stool, and possible hernia.

Table S14. Routine biochemistry and hematology testing sample sizes by analyte, timepoint, and intervention group.

Analyte	Age (days)[a]	Intervention Group				
		Placebo, N	LP1, N	LP1+FOS, N	LP7, N	LP7+FOS, N
Procalcitonin	9	35	44	39	44	38
	60	56	46	52	47	50
High-sensitivity CRP	9	35	44	39	44	38
	60	56	46	53	47	50
Creatinine	9	35	44	38	43	38
	60	56	46	53	47	50
Alanine transaminase	9	35	44	39	44	38
	60	56	46	53	47	50
Total bilirubin	9	35	44	39	44	38
	60	56	46	53	47	50
Direct bilirubin	9	35	44	39	44	38
	60	56	46	53	47	50
Indirect bilirubin	9	35	44	39	44	38
	60	55	46	53	47	50
Albumin	9	35	44	38	43	38
	60	56	46	53	47	50
Glucose	9	33	43	37	43	37
	60	56	46	53	47	50
Hemoglobin	9	34	43	39	44	38
	60	56	46	52	47	49
Hematocrit	9	34	43	39	44	38
	60	56	46	52	47	49
Red blood cells	9	34	43	39	44	38
	60	56	46	52	47	49
Mean corpuscular volume	9	34	43	39	44	38
	60	56	46	52	47	49

Mean corpuscular hemoglobin	9	34	43	39	44	38
	60	56	46	52	47	49
Total leukocyte count	9	34	43	39	44	38
	60	56	46	52	47	49
Neutrophils	9	34	43	39	44	38
	60	56	46	52	47	49
Lymphocytes	9	34	43	39	44	38
	60	56	46	52	47	49
Monocytes	9	34	43	39	44	38
	60	56	46	52	47	49
Eosinophils	9	34	43	39	44	38
	60	56	46	52	47	49
Basophils	9	34	43	39	44	38
	60	56	46	52	47	49
Platelet count	9	34	43	39	44	38
	60	56	46	52	47	49

[a]Median (25th, 75th) age at sample collection for routine day 9 and 60 samples was 9 (9, 11) and 61 (60, 66), respectively.

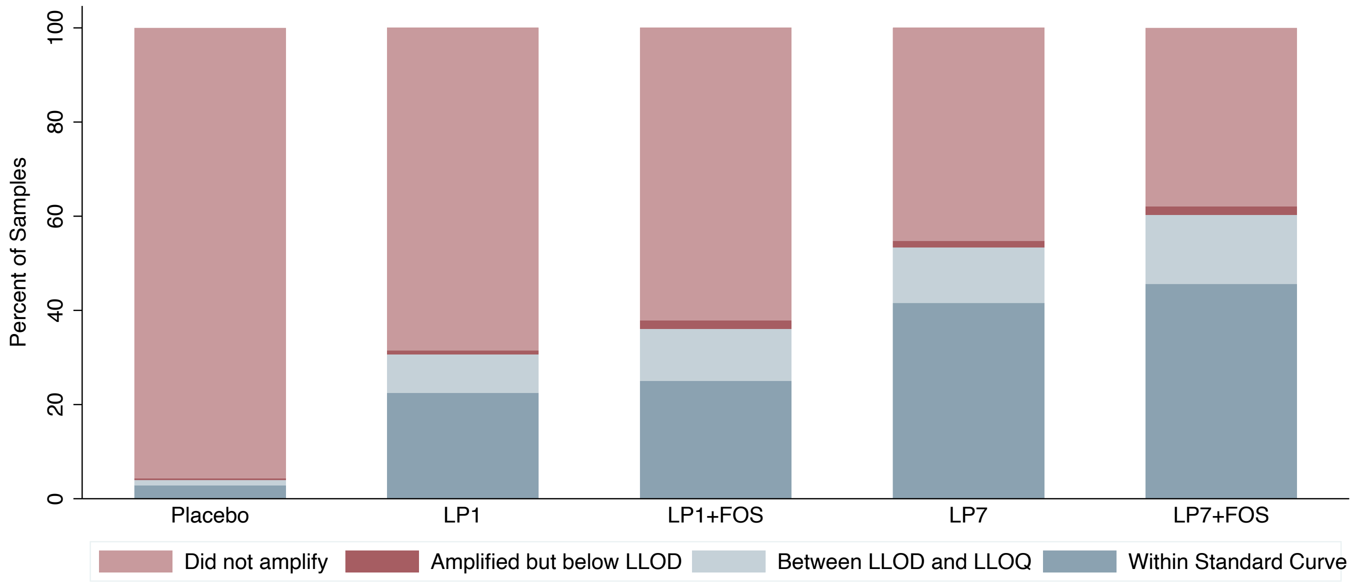

Figure S1. Distribution, by intervention group, of amplification status of infant stool samples collected post-intervention (days 14 to 60) using a qPCR assay that was optimized for detection of LP202195. The proportion of all post$_{14\text{-}60}$ stool samples (n=1393) that either did not amplify (shaded light maroon), amplified with a cycle quantification (Cq) value below the assay's lower limit of detection (LLOD) (shaded dark maroon), amplified with a Cq value between the LLOD and lower limit of quantification (LLOQ) (shaded in light blue), or amplified with a Cq value that fell within the standard curve (shaded dark blue) are shown by intervention group.

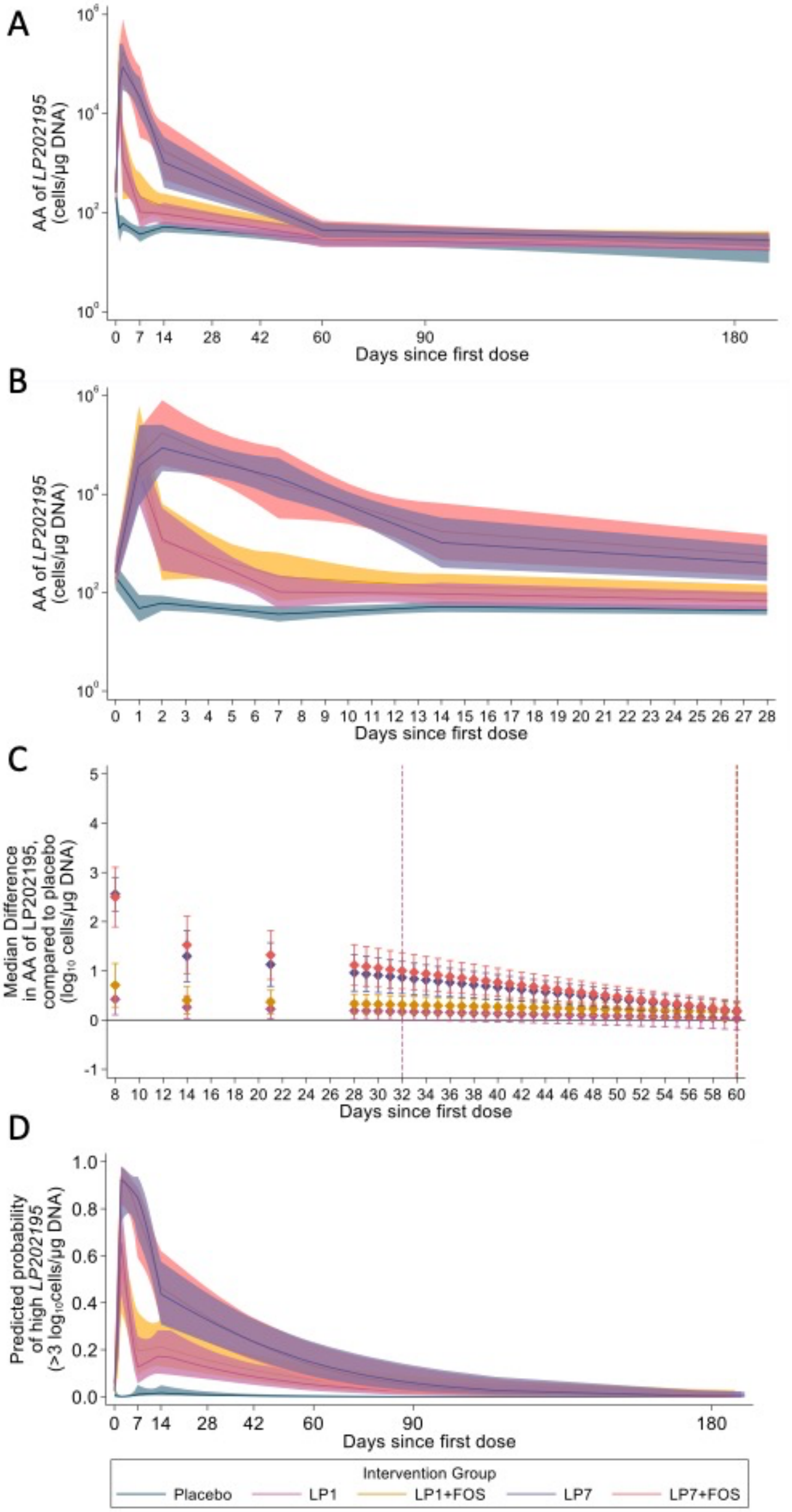
A
AA of LP202195 (cells/μg DNA)
Days since first dose
B
AA of LP202195 (cells/μg DNA)
Days since first dose
C
Median Difference in AA of LP202195, compared to placebo (log10 cells/μg DNA)
Days since first dose
D
Predicted probability of high LP202195 (>3 log10cells/μg DNA)
Days since first dose
Intervention Group
Placebo
LP1
LP1+FOS
LP7
LP7+FOS

Figure S2. *L. plantarum* ATCC 202195 (LP202195), when administered to neonates in Dhaka, Bangladesh for 1 or 7 days, with or without fructooligosaccharide (FOS), does not result in LP202195 colonization. This figure is the same as Figure 2 in the main manuscript except for the addition of 95% confidence interval (95%CI) shading. A) Longitudinal trajectories of LP202195 absolute abundance (AA) (cells/µg DNA) modelled as a function of days since first IP dose, up to 180 days since first dose, and B) zooming in on the trajectories within the first 28 days since first IP dose. The shading in panels A and B represents the 95%CIs of the predicted median AAs. C) Differences in AA of LP202195 (log_{10} cells/µg DNA) across intervention groups, relative to the placebo group, are shown at discrete time points between 8 and 60 days since first IP dose. The vertical dashed blue line at 32 days since first dose indicates the time point at which the AA in the LP1 group was no longer significantly different from the AA in the placebo group. The vertical dashed red line at 60 days since first dose indicates the time point at which the AAs LP1+FOS, LP7, and LP7+FOS groups were no longer significantly different from the AA in the placebo group. D) Predicted probabilities of high versus low AA of LP202195, by days since first dose. The shading represents the 95%CIs of the predicted probability of a high AA. In each panel, the placebo group is shown in blue, the LP1 group in pink, the LP1+FOS group in yellow, the LP7 group in purple, and the LP7+FOS group in red.
